# Intrinsically Disordered Polypeptides‐Based Stealth Materials Enable Enhanced Photothermal Cancer Therapy Using an In Situ Fiber‐Based Penetrating Laser System

**DOI:** 10.1002/smsc.70342

**Published:** 2026-07-14

**Authors:** Kei Nishida, Hiromasa Yamashita, Akira Takahashi, Eijiro Miyako

**Affiliations:** ^1^ Graduate School of Advanced Science and Technology Japan Advanced Institute of Science and Technology Nomi Ishikawa Japan; ^2^ Wellness Development Center AIR WATER INC. Tokyo Japan; ^3^ Advanced Comprehensive Research Organization Teikyo University Tokyo Japan; ^4^ Institute of Multidisciplinary Research for Advanced Materials Tohoku University Sendai Miyagi Japan; ^5^ Department of Biomolecular Engineering, Graduate School of Engineering Tohoku University Sendai Miyagi Japan; ^6^ Advanced Research Center for Innovations in Next‐Generation Medicine Tohoku University Sendai Japan

**Keywords:** carbon nanohorn, contact‐mode laser irradiation system, endoscope, intrinsically disordered polypeptides, photothermal cancer therapy

## Abstract

Photothermal therapy has emerged as a minimally invasive cancer treatment strategy, yet poor nanoparticle (NP) stability, nonspecific accumulation, and inefficient laser delivery to deep tumor tissues markedly limit conventional approaches. Here, we report the rational design of intrinsically disordered polypeptides (IDPs) as next‐generation stealth materials for photothermal cancer therapy applications using a novel contact‐mode laser irradiation system. IDP1 was computationally designed as a hydrophilic IDP inspired by albumin’s primary sequence. IDP1 exhibited an exceptional fourfold prolonged blood circulation half‐life (*t*_1/2_ = 150.5 min) relative to PEG (*t*_1/2_ = 17.8 min). IDP1‐conjugated lipid NPs encapsulating carbon nanohorns and indocyanine green (IDP1‐CNH/ICG) demonstrated efficient tumor accumulation via the enhanced permeability and retention effects. We developed a graded‐index plastic optical fiber endoscope system with integrated real‐time fluorescence imaging capability, enabling image‐guided contact‐mode near‐infrared laser irradiation and achieving superior photothermal conversion compared to conventional noncontact irradiation, representing 27% higher heating efficiency. In Colon26‐bearing mice, complete tumor regression occurred within 14 days after single‐dose IDP1‐CNH/ICG administration combined with contact‐mode laser irradiation, without recurrence or systemic toxicity. Overall, this study establishes IDP1 as versatile stealth biomaterials and demonstrates the synergistic potential of advanced NP design with innovative laser delivery systems for effective cancer therapy.

## Graphical abstract

Rationally designed intrinsically disordered protein‐conjugated lipid nanoparticles encapsulating carbon nanohorns achieve superior tumor accumulation and complete cancer regression via a novel contact‐mode fiber‐optic laser delivery system, establishing a new paradigm for effective photothermal theranostics.

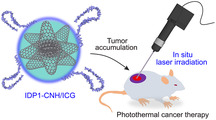

## 1 Introduction

Photothermal therapy (PTT) represents a promising minimally invasive approach for cancer treatment, utilizing near‐infrared (NIR) light‐excitable photothermal agents that convert optical energy into localized heat with high efficiency [1, 7]. NIR irradiation within biological windows (650–950 and 1000–1350 nm) offers several advantages: deeper tissue penetration (up to several centimeters), minimal autofluorescence, and reduced collateral damage to surrounding healthy structures compared to visible light. NIR exposure enables photothermal agents to elevate local temperatures to therapeutic ranges (typically 42–50 °C), triggering heat‐mediated cancer cell death through multiple mechanisms, including apoptosis, necrosis, and immunogenic cell death. Through vascular disruption, protein denaturation, and stress‐mediated immune modulation, photothermal heating potentiates antitumor activities [8, 12].

Carbon nanohorns (CNH), single‐walled carbon nanostructures with unique horn‐shaped tips, exhibit exceptional photothermal conversion properties in the NIR region, excellent photostability, and resistance to photobleaching [13, 16]. However, CNH intrinsic hydrophobicity, aggregation propensity, and serum protein–CNH interactions severely limit CNH physiological dispersibility, necessitating sophisticated nanoparticle (NP) formulations. Our previous work demonstrated that lipid NPs comprising CNH and polyethylene glycol (PEG)‐conjugated phospholipids serve as effective platforms for PTT, achieving selective tumor accumulation via the enhanced permeability and retention (EPR) effect and inducing selective cytotoxicity upon irradiation [17]. Other carbon nanomaterials, including carbon nanotubes and graphene derivatives, have also been extensively investigated as photothermal agents because of their strong NIR absorption and high photothermal conversion efficiencies [5]. However, CNHs offer several advantages for biomedical applications, including metal‐catalyst‐free synthesis, lower concerns regarding residual catalyst toxicity, unique dahlia‐like aggregate structures facilitating NP formulation, and excellent photostability. These characteristics make CNHs particularly attractive for constructing stable photothermal nanoplatforms.

Despite widespread use, PEG‐based stealth systems face several emerging limitations. Anti‐PEG antibodies generated upon repeated administration can accelerate blood clearance and perturb PEGylated NP biodistribution through the “accelerated blood clearance (ABC) phenomenon.” [18, 21] Moreover, PEGylated NPs still exhibit substantial uptake by macrophages due to protein corona formation at particle interfaces, leading to preferential accumulation in reticuloendothelial organs (liver and spleen) rather than tumor tissue [22, 26]. Therefore, alternative stealth materials with superior antifouling properties, enhanced circulation longevity, and improved biocompatibility need to be developed.

Polypeptide‐based stealth materials represent promising alternatives, as they combine inherent biocompatibility and biodegradability with tunable physicochemical properties arising from amino acid compositional diversity [27, 29]. Among these, XTEN polypeptides—designed as stealth sequences comprising six amino acids (alanine, glutamate, glycine, proline, serine, and threonine)—form hydrophilic unstructured chains‐like stealth behavior and enable half‐life extension of therapeutic proteins [30, 31]. Stealth properties of XTEN derived from steric repulsion within its molecular chain were reflected in a strong anionic charge and long nonstructured chain (864 amino acids), relating to decreased isoelectric point (pI) values and conjugated molecule potency [32]. Optimized polypeptide‐based stealth molecule design remains an active area that requires further innovation.

Beyond NP optimization, effective PTT also depends critically on efficient laser delivery to tumor tissues. Conventional noncontact laser irradiation systems suffer from substantial light attenuation through tissue layers, limiting penetration depth and necessitating high laser powers that risk thermal damage to surrounding healthy tissues. This fundamental limitation has prompted the exploration of alternative delivery strategies. Interstitial laser irradiation using optical fibers inserted directly into tumors has shown promise in clinical applications [33, 37], yet existing systems lack the miniaturization and precision needed for small animal models and minimally invasive procedures. Direct contact‐mode laser delivery systems could overcome these limitations by minimizing the light path through healthy tissue, reducing required laser power, and enabling localized photothermal conversion with minimal collateral damage. However, such systems require careful engineering to balance efficiency, safety, and practical applicability. Furthermore, integration of real‐time imaging capabilities into such delivery systems would enable the visualization of therapeutic agent distribution, providing immediate feedback for optimal fiber positioning and validation of targeting efficacy prior to treatment initiation.

**FIGURE 1:**
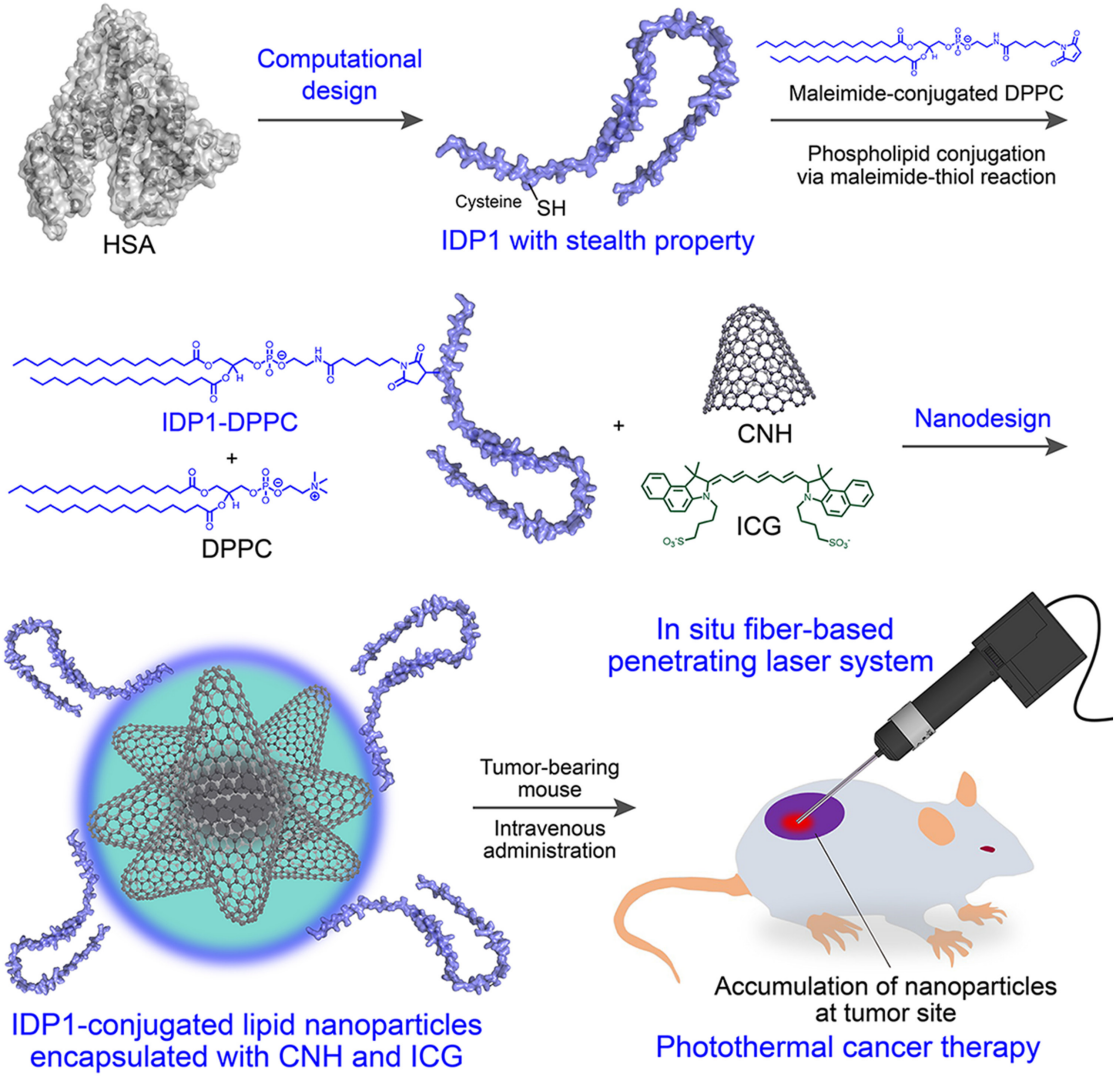
Conceptual overview of IDP1‐conjugated lipid NPs for photothermal cancer therapy using GI‐POF rigid endoscope systems enabling minimally invasive contact‐mode NIR laser irradiation directly to tumor tissue.

To address these dual challenges in NP design and laser delivery, we developed a comprehensive PTT platform combining rationally designed stealth polypeptides with an innovative contact‐mode laser irradiation system (Figure 1). Artificial intrinsically disordered polypeptides (IDPs) inspired by human serum albumin (HSA)—a widely used blocking agent—were then developed. Our computational design approach reconstructed albumin’s primary structure as hydrophilic IDP1 (∼100 residues from 11 amino acids) with enhanced blood retention time compared to XTEN and PEG. Phospholipid‐conjugated IDP1 and 1, 2‐dipalmitoyl‐sn‐glycero‐3‐phosphocholine (DPPC) facilitated the formation of stable CNH‐ and indocyanine green (ICG)‐containing lipid NPs with exceptional dispersion stability and low cellular internalization. Critically, we engineered a graded‐index plastic optical fiber (GI‐POF) rigid endoscope system [38, 42] with integrated fluorescence imaging capability, enabling image‐guided contact‐mode NIR laser irradiation to tumor tissue through a minimally invasive 16‐gauge (16G) needle insertion. Real‐time fluorescence monitoring during endoscope insertion confirmed IDP1‐CNH/ICG tumor accumulation and enabled optimized fiber positioning for maximal therapeutic efficacy. This novel approach overcomes the penetration limitations of conventional noncontact laser systems, enabling effective photothermal conversion at lower laser powers while minimizing damage to surrounding healthy tissue. The GI‐POF rigid endoscope represents a notable technological advancement over traditional fiber‐optic systems, offering superior flexibility, ease of handling, and compatibility with standard clinical needle gauges, thereby bridging the translational gap between preclinical studies and clinical applications. In Colon26‐bearing mice, IDP1‐CNH/ICG NPs combined with contact‐mode laser irradiation achieved complete tumor regression following a single treatment cycle, demonstrating the synergistic potential of advanced biomaterial design with innovative laser delivery technology. This integrated approach establishes a new paradigm for effective photothermal cancer therapy and highlights the broader applicability of IDP1 beyond oncology, including potential applications in biopharmaceutical half‐life extension and drug delivery.

## 2 Results and Discussion

### 2.1 Rational Design of IDP1

High flexibility and hydrophilicity represent common features of effective stealth materials, including polysaccharides, PEG, and zwitterionic polymers such as poly(carboxybetaine methacrylate) and poly(2‐methacryloyloxyethyl phosphorylcholine), which form excluded volume and hydration layers that resist protein adsorption [43, 44]. Among protein‐based antifouling agents, albumin is widely used as a blocking reagent to prevent nonspecific binding of target molecules to surfaces in biological assays. However, albumin is a folded globular protein that forms a dynamic protein corona with conformational changes upon surface adsorption, leading to unpredictable interfacial recognition and biodistribution [45]. We hypothesized that extracting the mechanistic origin of albumin’s blocking property—the presentation of water‐facing hydrophilic residues—into a flexible, unstructured polypeptide format would yield superior stealth materials.

**FIGURE 2:**
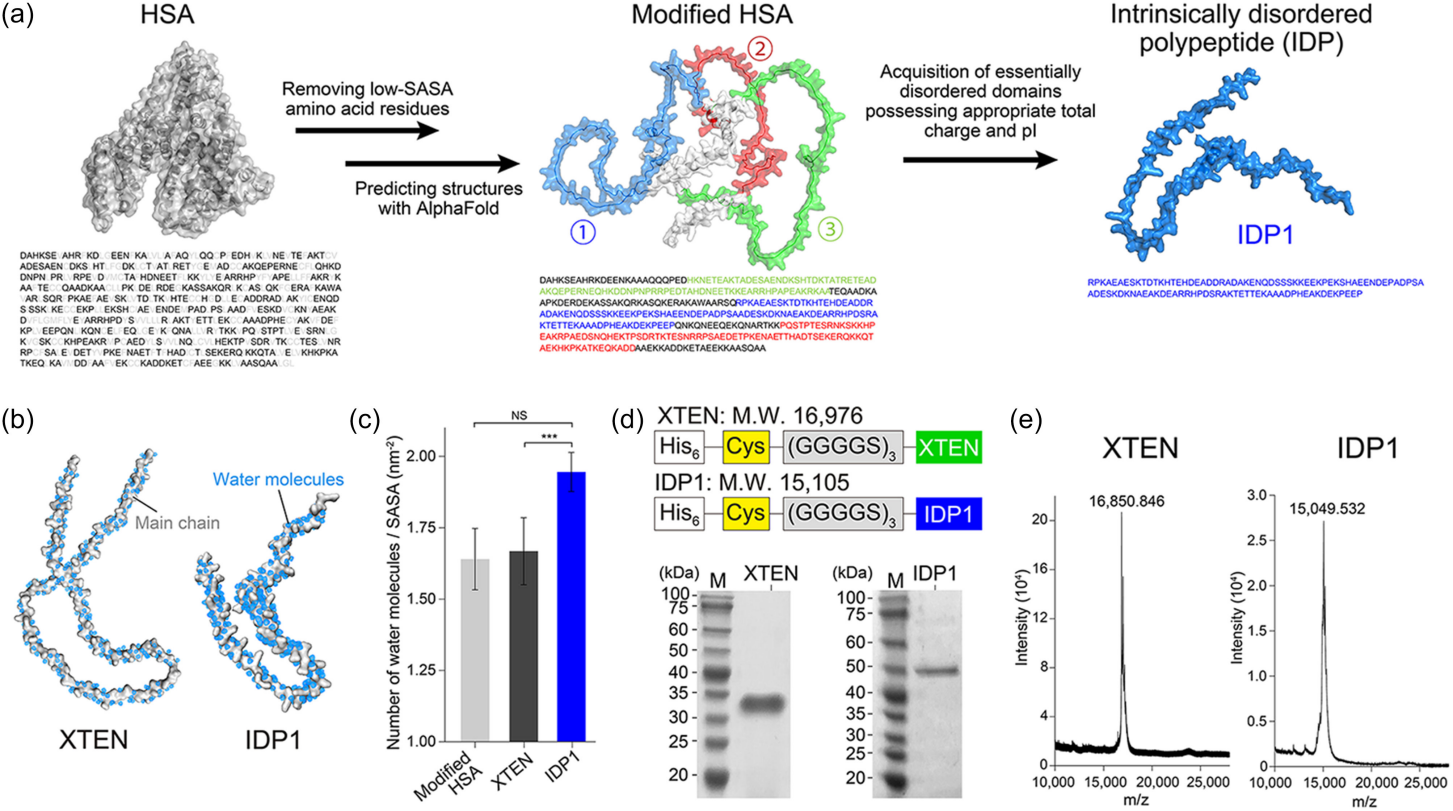
Rational design and characterization of artificial IDP1 derived from HSA. (a) Computational design scheme for IDP generation. (b) Visualization of hydrated water molecules (blue spheres) on XTEN and IDP1, calculated using a HydraProt deep learning tool [46]. Water molecules are shown as Gaussian density surfaces around protein backbones. (c) Quantitative comparison of the average hydration numbers on modified HSA, XTEN, and IDP1. Hydration numbers were calculated for five AlphaFold‐predicted structures and presented as mean values ± SDs. Statistical analysis by Tukey–Kramer multiple comparison test (***p < 0.001, NS: not significant; *n* = 5). (d) Modular structure and SDS‐PAGE analysis of recombinant XTEN and IDP1 polypeptides composed of an N‐terminal His_6_ tag for purification, a single cysteine (Cys) residue for conjugation chemistry, a flexible (GGGGS)_3_ linker to minimize steric interference, and the designed XTEN or IDP1 polypeptide sequence. The molecular weights (M.W.) shown correspond to the theoretical molecular weights of the full recombinant protein constructs, including the His_6_ tag, Cys residue, and linker sequence. M indicates the molecular weight marker in SDS‐PAGE. (e) MALDI‐TOF mass spectrometry profiles of IDP1 and XTEN.

To design stealth polypeptide sequences inspired by the HSA primary structure, we devised the following computational pipeline: (1) Calculate solvent‐accessible surface area (SASA) of each amino acid in HSA to identify hydrophilic residues; (2) Remove residues with SASA values ≤25% from the full‐length HSA sequence as hydrophobic residues, yielding modified HSA; (3) Predict the structure of modified HSA using AlphaFold to identify intrinsically disordered regions; (4) Extract candidate sequences from disordered regions and predict their total charge and pI to decide lead molecules (Figure 2a).

The average SASA value for each amino acid type was defined as its hydrophilicity index (Figure S1). Amino acids with high average SASA values interact frequently with solvent water, indicating surface exposure probability. Setting the threshold at 25% average SASA, amino acid residues below this value were eliminated from the HSA sequence as hydrophobic amino acids. The SASA value for native HSA was 28.9 × 10^3^ Å^2^, whereas modified HSA increased to 50.6 × 10^3^ Å^2^, suggesting substantially enhanced water interaction potential. From the AlphaFold‐predicted structure of the modified HSA, three candidates corresponding to intrinsically disordered regions were identified. Although the hydrophilic parameters of the three candidates showed strong negative values corresponding to hydrophilicity, total charges and pI calculations indicated that Candidate 2 exhibits more cationic character than Candidates 1 and 3 (Table 1). Cationic polypeptides strongly interact with negatively charged proteins and the plasma membrane of immune cells in blood, leading to poor stealth properties [47, 50]. In contrast, Candidate 3 showed a relatively neutral charge sequence compared to Candidate 1, indicating lower colloidal stability in physiological environments. Therefore, we selected Candidate 1 with a weak anionic polypeptide as IDP1, leading to the high flexibility and hydrophilicity required for stealth properties.

**TABLE 1:** Physicochemical characterization of IDP candidates and XTEN control.

Code	Amino acids	Calculated molecular weight of designed polypeptide domain	Total charges	**pI** a	**Hydrophilic parameter** b
Candidate 1 (IDP1)	102	11,400	−9	5.41	−57.9
Candidate 2	88	10,900	5	8.97	−61.4
Candidate 3	80	9,200	0	6.45	−51.2
XTEN	144	13,200	−24	3.90	−6.13

a Theoretical isoelectric point calculated using ExPASy ProtParam tool based on amino acid sequence.

b Hydrophilic parameter calculated as the sum of individual amino acid hydrophobicity values, which was the consensus scale by Eisenberg et al. [47]. More negative values indicate higher hydrophilicity.

Molecular chain hydration is a key parameter governing stealth properties [43, 44, 51, 52]. The hydration number of IDP1 was calculated using HydraProt, a deep learning tool developed by Zamyatnin et al. for predicting water molecule positions around protein structures [46]. Although predicting water distribution around highly disordered proteins remains challenging, HydraProt provides preliminary estimates for comparison. The XTEN with a molecular weight comparable to IDP1 was used as a control sample (Figure S2). The number of hydrated water molecules around the molecular chain ranked as IDP1 > XTEN (Figure 2b,c), suggesting that IDP1 possesses enhanced hydration capacity relative to conventional stealth polypeptides.

To evaluate the experimental stealth properties, IDP1 were produced using an *E. coli* expression system. Cysteine residues were introduced at the N‐terminus as reactive functional groups for fluorescent labeling or lipid modification (Figure 2d). XTEN were constructed as control materials. SDS‐PAGE analysis revealed unimodal bands corresponding to IDP1 and XTEN, although each migrated at apparent molecular weights higher than theoretical values. This anomalous migration behavior reflects weak SDS binding to polypeptides rich in hydrophilic amino acids, a characteristic feature of IDP1. Molecular weights were therefore confirmed by MALDI‐TOF/MS (Figure 2f–h). Mass spectra exhibited single dominant peaks at m/z values matching the calculated theoretical masses of IDP1: 15 105 and XTEN: 16 976, confirming successful synthesis of the designed IDP1.

### 2.2 Comprehensive Evaluation of Stealth Properties of IDP1

**FIGURE 3:**
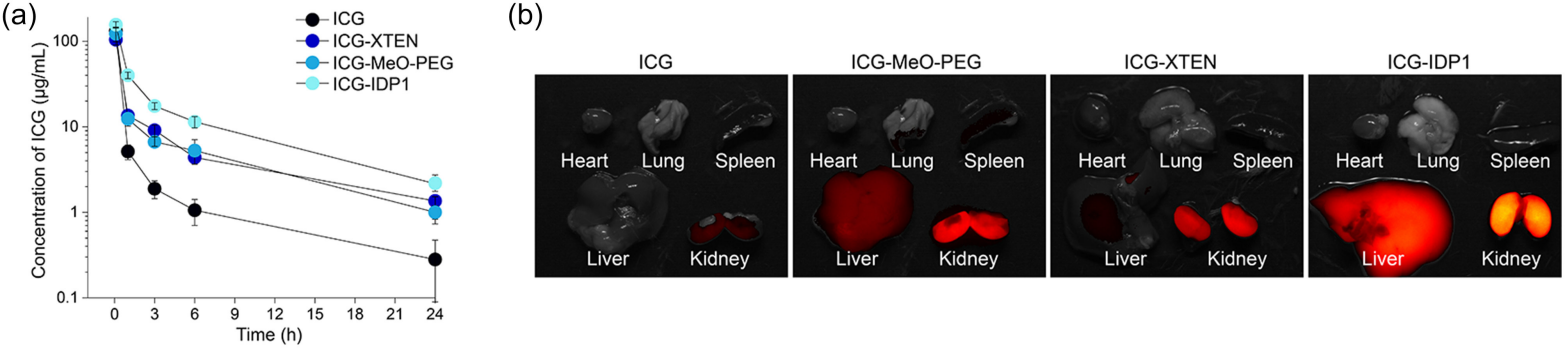
Comprehensive evaluation of stealth properties of IDP1. (a) Intravenous administration (200 μL, 0.1 mg mL^−1^ ICG). Blood samples were collected at the indicated time points, and plasma ICG concentration was determined by fluorescence intensity. Data are presented as mean ± SD (*n* = 4 mice per group). (b) Representative in vivo fluorescence images of major organs 24 h post‐administration. Images acquired using an NIR fluorescence imaging system (excitation 740–790 nm, emission 810–860 nm, 3 s exposure).

To clarify stealth properties of IDP1, the plasma concentration profiles of intravenously administered IDP1 in mice were investigated (Figure 3a). For the fluorescence‐based evaluation, IDP1 was conjugated with an NIR fluorescence dye (maleimide‐modified ICG) via a thiol‐maleimide reaction to generate ICG‐IDP1 (See Supporting Information). Monomethoxy PEG (*M*_w_ 5,000, MeO‐PEG) with a similar chain length served as a control. ICG‐IDP1 markedly prolonged the plasma ICG concentration compared to ICG alone, XTEN, and MeO‐PEG. The elimination half‐life (*t*_1/2_) of ICG‐IDP1 was 150.5 min, whereas *t*_1/2_ values for ICG alone, ICG‐XTEN, and ICG‐MeO‐PEG were 3.9, 35.7, 34.5, and 17.8 min, respectively, representing a fourfold improvement over PEG and >eightfold improvement over XTEN.

The systemic distribution of intravenously administered IDP1 was assessed using in vivo fluorescence imaging. At 24 h post‐administration, ICG‐IDP1 had higher fluorescence intensity in the liver and kidney than ICG‐XTEN and ICG‐MeO‐PEG, while ICG alone was minimally detected (Figure 3b). Prolonged organ retention of ICG‐IDP1 relative to ICG‐XTEN and ICG‐MeO‐PEG suggests delayed renal and hepatic clearance. Considering that XTEN exhibited comparable *t*_1/2_, the greater organ retention observed for IDP1 indicates enhanced resistance to proteolytic degradation and slower clearance relative to XTEN. Although detailed mechanisms require further investigation, IDP1’s combination of high chain flexibility and hydrophilicity confers superior stealth properties, including dramatically prolonged circulation half‐life, a critical parameter for effective drug delivery and tumor accumulation. The superior circulation longevity of IDP1 may partly originate from reduced plasma protein adsorption arising from its highly hydrated and flexible molecular chain. Although direct protein corona characterization was beyond the scope of the present study, future quantitative proteomic analyses will provide mechanistic insights into the antifouling behavior of IDP1. Although IDP1 exhibited substantially prolonged circulation compared with PEG after a single administration, the potential avoidance of ABC following repeated administration remains to be investigated. Future studies will evaluate anti‐IDP1 immune responses and pharmacokinetic behavior after multiple dosing cycles.

### 2.3 IDP1‐Based Lipid NPs Incorporating CNH

Having established IDP1’s superior stealth properties, we synthesized phospholipid‐conjugated IDP1 and fabricated lipid NPs incorporating CNHs as photothermal agents (See Supporting Information). Maleimide‐modified DPPC was conjugated to IDP1’s thiol group, yielding DPPC‐IDP1. NPs were formed from DPPC, DPPC‐IDP1, and CNH by sonication. DPPC‐IDP1 and DPPC‐MeO‐PEG markedly improved CNH water dispersibility compared to CNH alone or DPPC (Figure S3a,b). No CNH aggregation or absorbance decrease was observed over 3 days. Dynamic light scattering (DLS) measurements showed that DPPC‐IDP1‐composed NPs maintained particle sizes of approximately 80 nm over 3 days, whereas DPPC and DPPC‐PEG exhibited increased particle sizes due to precipitation and aggregation (Figure S3c,d). The stability evaluations performed in PBS demonstrated excellent colloidal stability. Further investigations under various serum concentrations will be valuable for assessing long‐term stability under physiologically relevant conditions.

**FIGURE 4:**
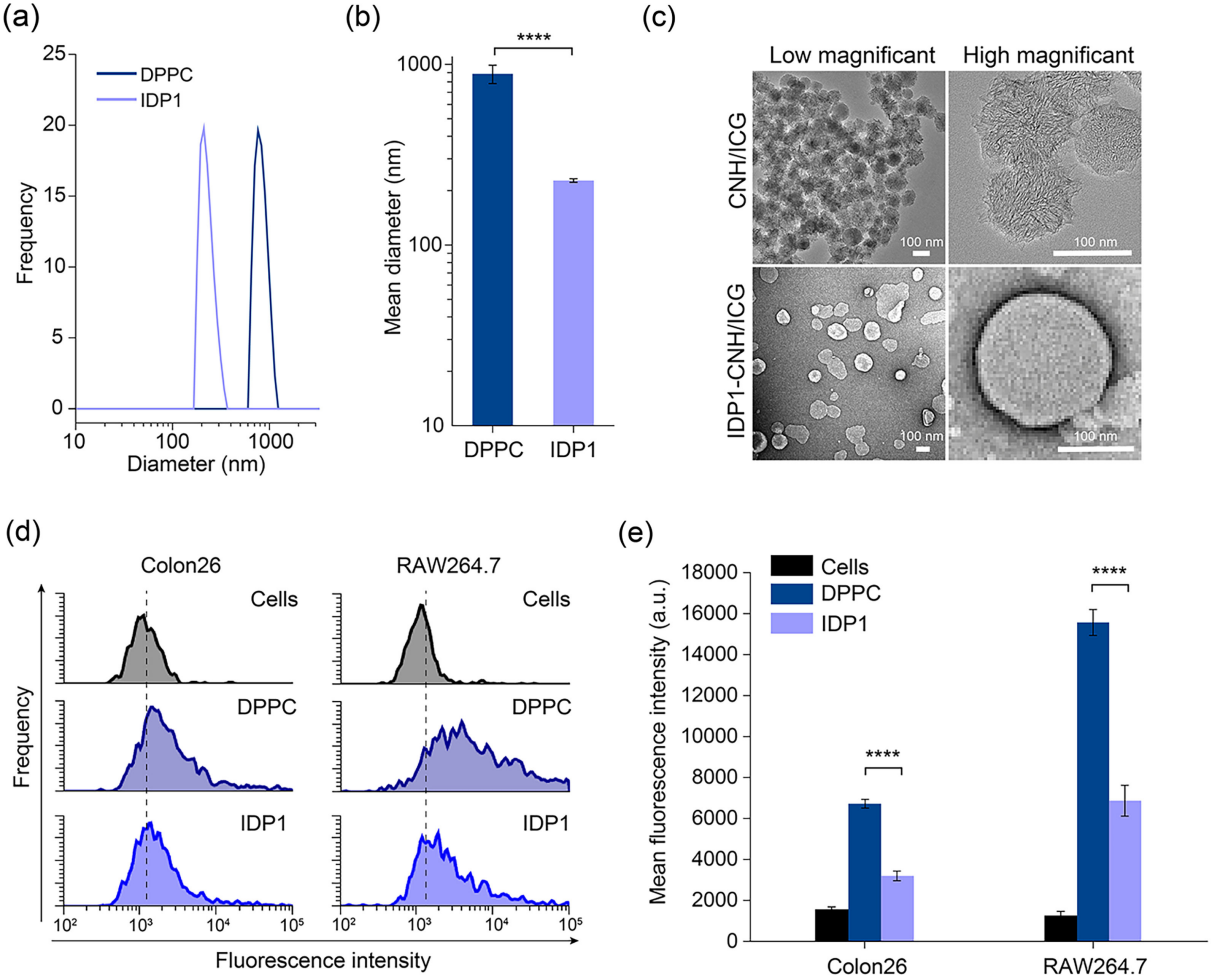
Lipid NPs composed of IDP1‐conjugated lipids and carbon nanohorn (CNH) with indocyanine green (ICG). (a) DLS size distribution profiles of DPPC‐CNH/ICG (control, without IDP1) and IDP1‐CNH/ICG NPs. (b) Average hydrodynamic diameters quantified from the DLS measurements. Data are presented as mean ± SD (*n* = 3 independent preparations). Statistical significance by Tukey–Kramer multiple comparison test (****p < 0.0001). (c) TEM images of CNH/ICG and IDP1‐CNH/ICG NPs. (d) Representative fluorescence histograms in flow cytometry for Colon26 cancer cells (left panels) and RAW264.7 macrophages (right panels). Cells were treated with DPPC‐CNH/ICG or IDP1‐CNH/ICG (10 μg mL^−1^ CNH/ICG) for 6 h. (e) Quantification of mean fluorescence intensity of cells treated with DPPC‐CNH/ICG or IDP1‐CNH/ICG (10 μg mL^−1^ CNH/ICG) for 6 h. Data are presented as mean ± SD (*n* = 3 independent experiments). Statistical significance by Tukey–Kramer multiple comparison test (****p < 0.0001).

For enhanced PTT, we incorporated CNH and ICG into DPPC‐IDP1‐composed NPs, as the combined CNH/ICG systems reportedly show strong local hyperthermia and cell death induction (Figure 4a,b) [17, 53]. DPPC‐IDP1 NPs exhibited sizes of approximately 200 nm, whereas DPPC alone formed larger aggregates (∼800 nm), demonstrating that DPPC‐IDP1 improved CNH/ICG dispersion through IDP1’s hydration and flexibility. Furthermore, IDP1‐CNH/ICG morphology was confirmed by transmission electron microscopy (TEM) analysis (Figure 4c). CNH/ICG morphology showed aggregates of typical dahlia‐like structures. In contrast, the micrographs revealed that IDP1‐CNH/ICG formed dispersed spherical particles with 155 ± 54.4 nm (Feret diameter). These results suggest that DPPC‐IDP1 dispersed CNH/ICG and formed stable NPs.

The cellular internalization of IDP1‐CNH/ICG into mouse colon tumor Colon26 cells and RAW264.7 macrophages was assessed by flow cytometry, detecting ICG fluorescence (Figure 4c,d). Colon26 and RAW264.7 cells treated with IDP1‐CNH/ICG showed significantly lower fluorescence intensity relative to DPPC‐CNH/ICG, indicating that IDP1’s stealth properties effectively suppressed NP internalization into both cancer cells and macrophages. This reduced cellular uptake, particularly by RAW264.7 macrophages, suggests decreased recognition by the reticuloendothelial system. Such suppression of macrophage uptake may contribute to prolonged blood circulation, thereby increasing the probability of passive tumor accumulation through the enhanced permeability and retention (EPR) effect.

To further investigate the intracellular behavior of the NPs, lysosomal colocalization studies were performed using LysoTracker staining. As shown in Figure S4, IDP1‐CNH/ICG NPs exhibited markedly lower intracellular fluorescence signals than DPPC‐CNH/ICG NPs in both Colon26 and RAW264.7 cells, indicating reduced cellular internalization. This observation is consistent with the reduced cellular uptake and macrophage internalization shown in Figure 4 and further supports the stealth properties of the IDP1 conjugation.

For the fraction of NPs that was internalized, substantial overlap between NP‐associated ICG fluorescence and lysosomal signals was observed, suggesting localization within endo/lysosomal compartments following uptake. These findings indicate that internalized NPs undergo conventional endocytic trafficking pathways. Although lysosomal escape is often desirable for intracellular drug delivery systems, it is not essential for the present PTT platform because therapeutic efficacy is achieved through local heat generation following NIR laser irradiation rather than intracellular cargo release.

### 2.4 Photothermal Conversion Analysis Using Contact‐Mode NIR Laser Irradiation

**FIGURE 5:**
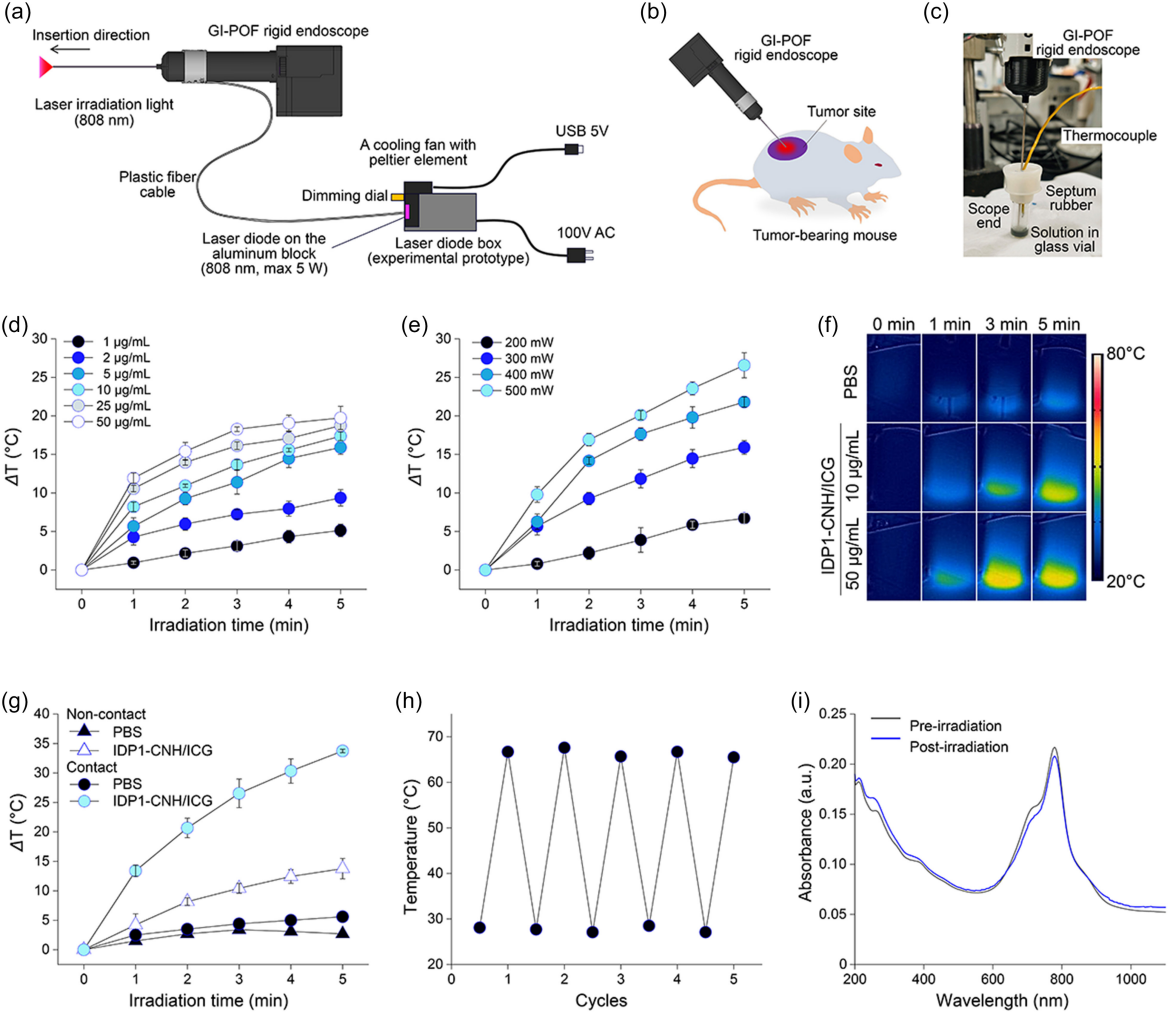
Photothermal conversion analysis using novel contact‐mode NIR laser irradiation via GI‐POF rigid endoscope. (a) Schematic diagram of the graded‐index plastic optical fiber (GI‐POF) rigid endoscope system architecture. The output power at the tip of the GI‐POF rigid endoscope was set to 300 or 500 mW, corresponding to power densities of approximately 91 and 152 W cm^−2^, respectively, based on a laser emission area of 0.33 mm^2^. (b) Illustration demonstrating minimally invasive contact‐mode laser irradiation to tumor‐bearing mice. (c) Experimental setup for photothermal conversion measurement in solution. (d) Concentration‐dependent photothermal heating of IDP1‐CNH/ICG suspension. Temperature increase (Δ*T*) was measured at CNH/ICG concentrations ranging from 1 to 100 μg mL^−1^ under constant 300 mW laser irradiation for 5 min. Data are presented as mean ± SD (*n* = 3). (e) Laser power‐dependent photothermal heating at a fixed IDP1‐CNH/ICG concentration (10 μg mL^−1^). Data are presented as mean ± SD (*n* = 3). (f) Thermographic images visualizing spatial temperature distribution during contact‐mode laser irradiation. IDP1‐CNH/ICG suspension (10 μg mL^−1^, top row) and 50 μg mL^−1^ (bottom row) were irradiated at 500 mW. (g) Direct comparison of contact‐mode versus noncontact‐mode laser irradiation efficiency. IDP1‐CNH/ICG suspension (10 μg mL^−1^) and PBS control were irradiated at 500 mW for 5 min using either contact‐mode (GI‐POF rigid endoscope insertion with power intensity ∼152 W cm^−2^) or noncontact‐mode (external 808 nm laser positioned ∼1 cm above solution surface). Data are presented as mean ± SD (*n* = 3). Power intensity at the fiber tip was calculated as 500 mW/0.33 mm^2^ = 152 W cm^−2^ based on a laser emission area of 0.33 mm^2^ at the endoscope tip. (h) Photothermal stability and recyclability assessment. IDP1‐CNH/ICG suspension (50 μg mL^−1^) was subjected to five repeated heating–cooling cycles (500 mW, 5 min heating followed by natural cooling to room temperature). (i) UV–vis‐NIR absorbance spectra of IDP1‐CNH/ICG before (pre‐irradiation, blue line) and after (post‐irradiation, red line) laser irradiation (500 mW, 5 min).

The photothermal conversion capability of IDP1‐CNH/ICG was evaluated under NIR (808 nm) laser irradiation. A novel GI‐POF rigid endoscope system was developed to deliver NIR laser light directly to the tumor tissue (Figure 5a,b). This GI‐POF rigid endoscope, equipped with a 0.5‐mm‐diameter lens and a coaxial plastic fiber bundle for NIR laser delivery, can be inserted into tissue through a 16G needle, enabling direct contact‐mode NIR laser irradiation. This approach represents a major technological advancement over conventional noncontact laser systems. Traditional external laser irradiation suffers from substantial light attenuation through overlying skin and tissue layers, typically requiring high laser powers (>1 W) that risk thermal damage to healthy tissues and skin burns. Existing interstitial fiber‐optic systems, while enabling internal tumor irradiation, generally use larger‐diameter fibers requiring more invasive insertion procedures. Our GI‐POF rigid endoscope overcomes these limitations through miniaturization compatible with standard clinical needle gauges, superior flexibility facilitating navigation through tissue, and efficient light transmission enabling therapeutic temperatures at lower power settings. The contact‐mode delivery mechanism minimizes the light path through healthy tissue, concentrates photothermal energy at the tumor site, and permits precise spatial control of thermal dose distribution.

The photothermal conversion capability of IDP1‐CNH/ICG was first measured in solution using the GI‐POF rigid endoscope with thermocouple temperature monitoring (Figure 5c). The IDP1‐CNH/ICG suspension in PBS showed a concentration‐dependent temperature increase (Δ*T*) under 300 mW laser irradiation (Figure 5d). At 10 μg mL^−1^ CNH/ICG concentration, Δ*T* increased with laser power (Figure 5e). Thermographic imaging confirmed photothermal conversion (Figure 5f), with visualization showing temperature increases consistent with thermocouple measurements.

Photothermal conversion efficiencies were compared between contact‐mode laser irradiation via a GI‐POF rigid endoscope and conventional noncontact‐mode irradiation (Figure 5g). At 500 mW laser power with a contact‐mode laser emission area of 0.33 mm^2^ at the endoscope tip (yielding a power intensity of approximately 152 W cm^−2^), contact‐mode irradiation of IDP1‐CNH/ICG suspension achieved Δ*T* = 36.3 °C after 5 min, whereas noncontact irradiation with 808 nm external laser achieved only Δ*T* = 28.5 °C, representing a 27% improvement in heating efficiency. Without IDP1‐CNH/ICG, both irradiation modes caused minimal temperature increases, confirming that heating derives from photothermal conversion rather than direct laser heating. Contact‐mode laser irradiation induced reproducible temperature increases over repeated heating–cooling cycles (Figure 5h). The UV–vis‐NIR absorption spectra of IDP1‐CNH/ICG remained unchanged after laser irradiation, demonstrating excellent photostability and suitability for repeated irradiation cycles (Figure 5i). Although photothermal conversion efficiency is commonly calculated under homogeneous bulk irradiation conditions, the present contact‐mode irradiation system generates highly localized heat through direct fiber‐mediated light delivery. Therefore, temperature elevation was used as the primary performance indicator because it more accurately reflects the practical therapeutic situation. These results establish that contact‐mode laser irradiation via a GI‐POF rigid endoscope achieves superior localized photothermal conversion using lower laser powers compared to conventional systems, potentially enhancing therapeutic efficacy while minimizing thermal damage to surrounding healthy tissues. For context, our previous CNH/ICG‐based nanoplatform under conventional noncontact NIR irradiation achieved a temperature elevation of Δ*T* ≈ 40 °C at 700 mW (808 nm, 5 min), despite a high intrinsic photothermal conversion efficiency of 63% [17]. Notably, the present contact‐mode system achieved a comparable Δ*T* of 36.3 °C at a 28% lower laser power of 500 mW, underscoring that irradiation geometry—rather than the intrinsic photothermal efficiency of the agent alone—is a decisive determinant of in situ heating performance. By eliminating light attenuation through overlying tissue layers, the GI‐POF rigid endoscope concentrates optical energy directly at the tumor site, enabling equivalent therapeutic heating at substantially reduced laser exposure. This reduction in required power is clinically significant, as lower laser doses minimize the risk of thermal damage to surrounding healthy tissues during interstitial treatment.

### 2.5 In Vitro and In Vivo Antitumor Efficacy of IDP1‐CNH/ICG

For in vitro evaluation, Colon26 cancer cells and human normal diploid fibroblast MRC5 cells treated with IDP1‐CNH/ICG suspension were irradiated with an NIR laser (300 mW, 2 min) using the GI‐POF rigid endoscope. At 24 h post‐irradiation, Colon26 cell viability decreased concentration‐dependently, whereas MRC5 viability remained largely unchanged (Figure S5). This differential cytotoxicity reflects the higher heat sensitivity of tumor cells than normal cells, consistent with our previous reports [17].

**FIGURE 6:**
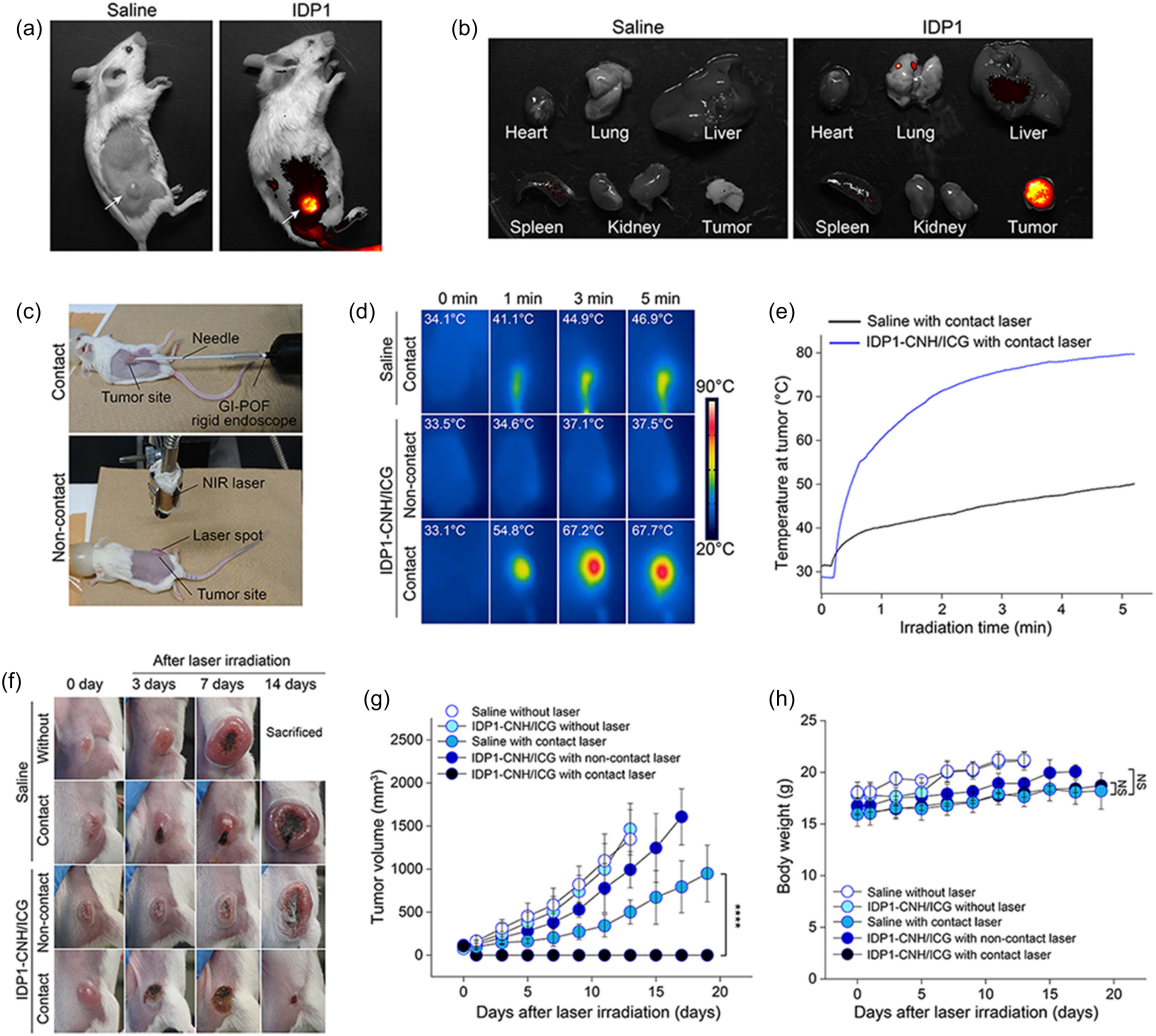
In vivo antitumor efficacy of IDP1‐CNH/ICG NPs with contact‐mode laser irradiation. (a) Whole‐body in vivo fluorescence imaging of Colon26 tumor‐bearing BALB/c mice at 24 h post‐intravenous administration of saline (left) or IDP1‐CNH/ICG (right, 200 μL, 100 μg mL^−1^ CNH/ICG) (Ex: 740–790 nm, Em: 810–860 nm). White arrows indicate tumor sites. (b) Ex vivo fluorescence imaging of major organs, heart, lung, liver, spleen, kidney, and tumors excised 24 h post‐administration. Images were acquired using the same acquisition parameters for direct comparison. Quantitative fluorescence intensity was determined by ROI analysis using CleVue software and expressed as mean ± SD (*n* = 3). (c) Experimental setup for in vivo PTT. Upper panel: Contact‐mode laser irradiation using a GI‐POF rigid endoscope inserted through a 16G needle. Lower panel: Noncontact‐mode laser irradiation using external 808 nm NIR laser positioned ∼1 cm above tumor surface. (d) Real‐time thermographic monitoring of the tumor surface temperature of saline‐administered and IDP1‐CNH/ICG‐administered mice during laser irradiation using contact‐mode laser and noncontact‐mode laser. Both modalities used 300 mW laser power and a 5 min irradiation duration. (e) Internal tumor temperature profile measured by thermocouple insertion during contact‐mode laser irradiation (300 mW). Saline‐treated (black line) and IDP1‐CNH/ICG‐treated tumors (blue line) were inserted with a thermocouple through a 16G needle. Data are presented as mean ± SD (*n* = 3 mice per group). (f) Representative photographs of tumor‐bearing mice during the treatment time course: (1) Saline without laser (control), (2) Saline with contact‐mode laser, (3) IDP1‐CNH/ICG with noncontact‐mode laser, (4) IDP1‐CNH/ICG with contact‐mode laser. (g) Quantitative tumor volume growth curves for tumor‐bearing mice. Data are presented as mean ± SD (*n* = 5 mice per group). Statistical significance assessed by Tukey–Kramer multiple comparison test (****p < 0.0001). (h) Body weight monitoring during the treatment period. All treatment groups maintained stable body weight with no significant differences, indicating absence of systemic toxicity from IDP1‐CNH/ICG administration or laser irradiation. Data are presented as mean ± SD (*n* = 5 mice per group, NS: not significant).

The in vivo anticancer therapeutic efficacy was investigated using a syngeneic Colon26 tumor model in BALB/c mice. IDP1‐CNH/ICG (200 μL, 100 μg mL^−1^ CNH/ICG) was intravenously injected into tumor‐bearing mice, and in vivo fluorescence imaging was performed 24 h post‐administration (Figure 6a). Strong ICG fluorescence was detected at the tumor sites in IDP1‐CNH/ICG‐administered mice. Ex vivo fluorescence imaging demonstrated preferential accumulation of IDP1‐CNH/ICG in tumors with minimal fluorescence signals in major organs (Figure 6b). Quantitative ROI analysis revealed that tumor fluorescence intensity (8542  ± 1,203 AU) was markedly higher than that of the liver (892  ± 7 AU), kidney (456 ± 2 AU), heart (124  ± 3 AU), spleen (356  ± 3 AU), and lung (1129  ± 11 AU), confirming efficient tumor accumulation and minimal off‐target distribution. The ROI analysis further supported the stealth characteristics of IDP1, demonstrating substantially higher fluorescence accumulation in tumors than in RES‐associated organs such as the liver and spleen. IDP1‐CNH/ICG accumulation in tumors was achieved via the EPR effect based on vascular leakage and impaired lymphatic drainage, facilitated by the ∼200 nm NP diameter [54, 56]. The minimal fluorescence in the liver and kidney suggests that IDP1‐composed lipid NPs effectively suppressed renal and hepatic clearance while preserving NP integrity in circulation.

IDP1‐CNH/ICG‐accumulated tumors were irradiated with an NIR laser using either a contact‐mode (GI‐POF rigid endoscope) or noncontact‐mode laser at 300 mW for 5 min (Figure 6c). The tumor surface temperature was monitored using thermographic imaging during irradiation (Figure 6d). In saline‐administered mice, contact‐mode laser irradiation elevated the tumor surface temperature to approximately 46.9 °C after 5 min. In contrast, IDP1‐CNH/ICG‐administered mice showed dramatic tumor surface temperature increases to approximately 67.7 °C after 5 min of contact‐mode laser irradiation. Importantly, the heated area in IDP1‐CNH/ICG‐treated tumors was extensive, suggesting efficient photothermal conversion and thermal propagation. Because the optical fiber was inserted directly into the tumor tissue through a 16G needle, laser exposure to surrounding skin was minimized. Although transient superficial scarring was observed around the insertion site, no severe ulceration or apparent damage to adjacent normal tissues was observed throughout the observation period. Noncontact‐mode laser irradiation (300 mW, 5 min) showed no significant temperature increase in the tumor surfaces. This dramatic difference demonstrates that contact‐mode laser delivery overcomes tissue light attenuation limitations, enabling efficient photothermal conversion at tumor sites. Temperature changes within tumors during contact‐mode laser irradiation were measured directly by inserting a thermocouple into the tumor tissue (Figure 6e). Internal tumor temperature increased immediately upon laser irradiation, reaching approximately 80 °C after 5 min, confirming deep penetrating photothermal effects throughout the tumor volume.

The therapeutic efficacy of IDP1‐CNH/ICG with contact‐mode laser irradiation was evaluated in Colon26‐bearing mice (Figure 6f,g). Tumors in mice administered IDP1‐CNH/ICG completely disappeared within 14 days following single contact‐mode laser irradiation, demonstrating remarkable therapeutic efficacy. Saline‐administered mice showed slight tumor growth suppression with a contact‐mode laser, possibly due to localized heating at the fiber contact site. However, mice administered IDP1‐CNH/ICG with noncontact laser irradiation and saline‐administered mice with contact‐mode laser failed to achieve tumor regression, highlighting the critical synergy between IDP1‐CNH/ICG NPs and contact‐mode laser delivery. Mouse body weight remained relatively constant across all treatment groups during the experimental period, indicating an absence of systemic toxicity (Figure 6h). Notably, IDP1‐CNH/ICG‐treated mice showed no tumor recurrence even 40 days post‐treatment (Figure S6), demonstrating durable therapeutic responses.

**FIGURE 7:**
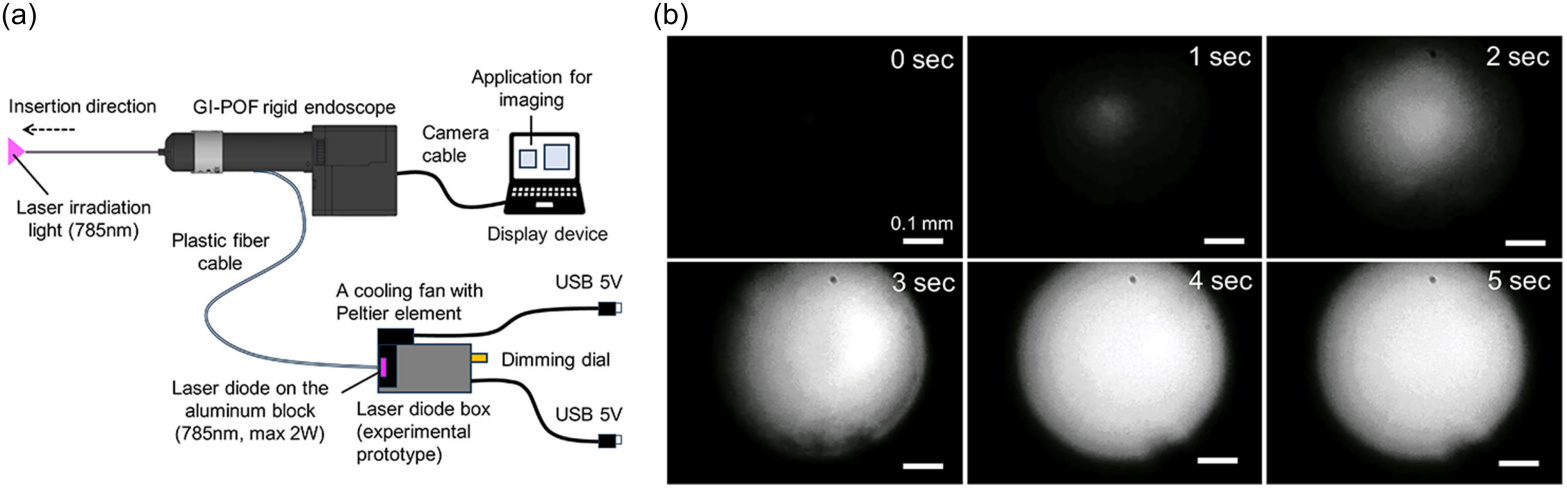
Real‐time fluorescence imaging capability of the GI‐POF rigid endoscope system for image‐guided PTT. (a) Schematic illustration of the integrated GI‐POF rigid endoscope system. The system comprises a 785 nm NIR laser module and a coaxial fluorescence imaging module (excitation 785 nm, emission 810–860 nm) for real‐time ICG visualization. The modular design enables image‐guided positioning of the fiber for optimal therapeutic delivery. (b) Representative real‐time fluorescence images captured during minimally invasive endoscope insertion into IDP1‐CNH/ICG‐accumulated tumors 24 h post‐administration. Fluorescence images reveal intense ICG distribution (white color) throughout the tumor volume. Time‐series images (0, 1, 2, 3, 4, and 5 s post‐insertion) confirm successful IDP1‐CNH/ICG accumulation and enable real‐time visualization for precise fiber positioning prior to laser irradiation. This image‐guided approach validates targeting efficacy and enables optimization of the laser delivery location.

The GI‐POF rigid endoscope system incorporates an integrated fluorescence imaging module that enables real‐time visualization of NP distribution during minimally invasive insertion (Figure 7a). The coaxial imaging system (excitation 785 nm, emission 810–860 nm) allows fluorescence imaging. Real‐time fluorescence monitoring during endoscope insertion into IDP1‐CNH/ICG‐accumulated tumors revealed intense ICG signals throughout the tumor volume (Figure 7b and Video S1), confirming successful NP targeting and enabling image‐guided fiber positioning at regions of highest accumulation prior to laser irradiation. This integrated imaging–therapy platform validates targeting efficacy in real time and enables optimized treatment delivery.

**FIGURE 8:**
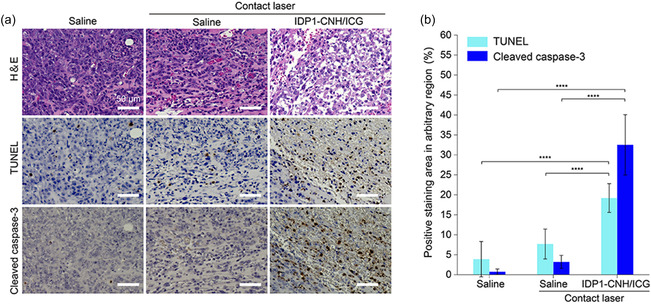
Histological and immunohistochemical analysis of PTT‐induced tumor damage. (a) Representative tissue sections from tumors collected at 6 h post‐laser irradiation and stained with H and E, TUNEL, and cleaved caspase‐3 IHC. Scale bars: 50 μm. (b) Quantification of TUNEL‐positive and cleaved caspase‐3‐positive cells as a percentage of total cells in arbitrary regions (10 randomly selected high‐power fields per tumor, 3 tumors per group). Data presented as mean ± SD (*n* = 10 fields from 3 independent tumors). Statistical significance assessed by Tukey–Kramer multiple comparison test (****p < 0.0001).

To elucidate tumor suppression mechanisms, tumor tissues were analyzed with hematoxylin and eosin (H and E) staining and immunohistochemistry (IHC) at 6 h post‐treatment (Figure 8a,b). H and E staining of tumors treated with IDP1‐CNH/ICG and contact‐mode laser showed extensive damage, including nuclear shrinkage, cytoplasmic blurring, and coagulative necrosis, characteristic features of PTT‐induced cell death [8, 12]. IDP1‐CNH/ICG‐administered mice with contact‐mode laser exhibited markedly increased numbers of TUNEL‐positive and cleaved caspase‐3‐positive cells compared to saline‐administered controls, indicating robust apoptosis induction. These results demonstrate that efficient photothermal conversion by IDP1‐CNH/ICG induces apoptosis and necrosis in tumor tissue, contributing to effective in vivo antitumor activity.

The biocompatibility of IDP1 and IDP1‐CNH/ICG was assessed by H and E staining of tissues from mice administered with IDP1 (200 μL, 100 μg mL^−1^) and IDP1‐CNH/ICG (200 μL, 100 μg mL^−1^ CNH/ICG) for 7 days (Figure S5). These tissues showed no significant histological changes compared to the saline‐administered controls. Furthermore, IDP1 and IDP1‐CNH/ICG hematological toxicity in mice was confirmed by assessing the complete blood counts (CBC) of mice intravenously injected with saline, IDP1, and IDP1‐CNH/ICG for 7 days (Table S1). Major blood counts showed no statistically significant differences among the saline, IDP1, and IDP1‐CNH/ICG groups, indicating that neither IDP1 nor IDP1‐CNH/ICG caused statistically significant hematological toxicity in mice. These findings confirm that IDP1 and IDP1‐CNH/ICG possess excellent biocompatibility alongside their stealth properties. Collectively, IDP1‐CNH/ICG combined with contact‐mode laser irradiation achieved effective tumor accumulation and exceptional antitumor therapeutic effects through superior photothermal conversion for cancer treatment.

## 3 Conclusion

This study reports the rational design of IDP1 as a next‐generation stealth material for photothermal cancer therapy applications using an innovative contact‐mode laser delivery system. Computational reconstruction of HSA yielded hydrophilic IDP1 (∼100 residues from 11 amino acids) with superior stealth properties. IDP1 exhibited a fourfold prolonged blood circulation half‐life compared to conventional PEG. IDP1 facilitated the formation of stable CNH‐containing lipid NPs that effectively accumulated in tumor tissue via the EPR effect.

We also developed a GI‐POF rigid endoscope system with integrated real‐time fluorescence imaging, enabling image‐guided contact‐mode NIR laser irradiation through minimally invasive needle insertion. This technological innovation overcomes the fundamental limitations of conventional noncontact laser systems, achieving 27% higher photothermal conversion efficiency at equivalent laser powers while minimizing damage to healthy tissues. The integrated imaging capability provides real‐time validation of NP tumor accumulation and enables optimized fiber positioning, bridging a critical translational gap between preclinical research and clinical implementation. In Colon26‐bearing mice, single‐dose IDP1‐CNH/ICG administration combined with contact‐mode laser irradiation achieved complete tumor regression within 14 days without recurrence or systemic toxicity, demonstrating the synergistic potential of advanced NP design with innovative laser delivery technology.

Beyond PTT applications, this study establishes IDP1 as versatile biomaterials for diverse biomedical applications. The design strategy—extracting hydrophilic, disordered regions from naturally occurring proteins—can potentially be applied to any protein scaffold, offering vast compositional diversity for optimizing specific biological functions. Potential applications include imparting stealth properties to biopharmaceuticals for half‐life extension, drug delivery vehicles, and tissue engineering scaffolds. Collectively, the convergence of computationally guided biomaterial design, precision NP engineering, and minimally invasive device innovation demonstrated here charts a translatable path toward more effective and safer photothermal cancer therapy. More broadly, the computational strategy of extracting hydrophilic disordered domains from endogenous proteins introduces a generalizable design principle for next‐generation stealth nanomaterials, with implications extending well beyond PTT.

## 4 Materials and Methods

### 4.1 Design of IDP1

The HSA sequence and structure were obtained from PDB entry 1e78. The SASA of each amino acid in HSA was calculated using PyMOL (ver. 2.6.0a0). SASA values were plotted for each amino acid type, and average SASA values were calculated (Figure S1). Amino acid residues with SASA values ≤25% were removed from the full‐length HSA sequence to obtain modified HSA. The structure of the modified HSA was predicted using AlphaFold, and intrinsically disordered regions were identified to yield candidate 1, 2, and 3 sequences. The structures of these intrinsically disordered regions were predicted using AlphaFold. For the predicted structures, hydration numbers were calculated using HydraProt (config.initial_ca *p* = 0.03, config.final_ca *p* = 0.03). Visualization of hydrated water molecules was performed using PyMOL (set gaussian_resolution, 2.0, buffer = 3).

### 4.2 Expression and Purification of IDP

DNA fragments encoding IDP1 and XTEN were synthesized by FASMAC (Kanagawa, Japan) and incorporated into the pCold vector to obtain pCold‐His‐(GGGGS)_3_ ‐IDP/XTEN plasmids according to our previous methods [57, 58]. Plasmid‐transformed BL21(DE3) cells were cultured in LB medium containing 50 μg mL^−1^ ampicillin at 37 °C for 16 h. Pre‐culture was diluted 100‐fold into fresh LB medium and grown to OD_600_ = 0.5. After cooling on ice for 30 min, protein expression was induced by adding IPTG (1 mM final concentration), followed by incubation at 15 °C for 18 h with shaking at 130 rpm. Cells were harvested by centrifugation (8000 × g, 3 min, 4 °C), frozen at −80 °C, and lysed by sonication in Ni‐NTA buffer (50 mM Tris‐HCl, 150 mM NaCl, 1 mM DTT, 20 mM imidazole, pH 8.0). The cleared lysate was incubated with Ni‐NTA resin at 4 °C for 30 min. Resin was washed sequentially with buffer containing increasing imidazole concentrations (10, 20, and 50 mM), and the target protein was eluted with 500 mM imidazole. The eluate was dialyzed (molecular weight cut‐off (MWCO): 3,500) against phosphate‐buffered saline (PBS: 150 mM NaCl, 16 mM Na_2_HPO_4_, 4 mM NaH_2_PO_4_, pH 7.4) and filtered through 0.22 μm membranes. Protein concentration was determined using a Pierce BCA Protein Assay Kit, and samples were stored at −20 °C. The molecular mass of IDP1 and XTEN was measured by MALDI‐TOF/MS (Bruker, UltrafleXtreme). Samples dissolved in TA30 (30:70 = acetonitrile: 0.1% TFA in water) were mixed with 2, 5‐dihydroxybenzoic acid (20 mg mL^−1^) dissolved in TA30. IDP1: observed m/z 15,949.632, theoretical M.W. 15,105 for the full recombinant construct including the His_6_ tag, cysteine residue, and linker sequence. XTEN: observed m/z 14,525.171, theoretical M.W. 14,620 for the full recombinant construct including the His_6_ tag, cysteine residue, and linker sequence.

### 4.3 Preparation of Lipid NPs

IDP1‐CNH NPs were prepared by mixing 1 mg CNH (average diameter ≈80–100 nm; purity 95%; metal‐free, NEC Corporation, Tokyo, Japan) and DPPC (10 mg mL^−1^, 1 mL) dissolved in PBS (pH 7.4), followed by pulse‐type sonication (VCX‐600; amplitude 40%, pulse on/off = 2 s/2 s, Sonics, Danbury, CT, USA) for 10 min in an ice bath. DPPC‐IDP solution (0.3 mg mL^−1^, 9 mL) was added to the CNH solution, followed by pulse‐type sonication for 10 min in an ice bath to obtain IDP1‐CNH NPs (0.1 mg mL^−1^ CNH). For preparing IDP1‐CNH/ICG, 1 CNH and 1 mg ICG were mixed with DPPC and DPPC‐IDP1 solution. The NP suspensions were prepared by sonication and used directly without further purification, centrifugation, or filtration. The dispersion and loading efficiency of CNH and ICG were assessed by UV–vis‐NIR spectrometry (V‐730 BIO; Jasco, Tokyo, Japan). Particle size distributions were measured using a Litesizer 500 (Anton Paar GmbH, Graz, Austria) at 25 °C, recording scattering at 175°. IDP1‐CNH/ICG and CNH/ICG morphology and structure were observed under a high‐resolution TEM (JEM‐2100Plus, Japan Electron Optics Laboratory, Tokyo, Japan). TEM observations were performed by the Hanaichi UltraStructure Research Institute Co., Ltd. (Aichi, Japan). Because the NPs were prepared by direct sonication‐assisted dispersion without purification or separation steps, essentially all added CNH and ICG components were retained in the final formulation. Based on UV–vis‐NIR absorbance measurements before and after preparation, the material recovery was estimated to exceed 99%. All NP formulations were stored at 4 °C in PBS and showed no noticeable aggregation, particle size changes, or absorbance changes, as confirmed by DLS and UV–vis‐NIR spectroscopy (Figure S3) for at least 1 week. NPs used for in vivo studies were freshly prepared within 24 h prior to administration.

### 4.4 Flow Cytometry Analysis

RAW264.7 cells were seeded in 24‐well tissue culture dishes at 5 × 10^4^ cells well^−1^. The cells were treated with IDP1‐CNH/ICG and DPPC‐CNH/ICG (10 μg mL^−1^ CNH/ICG) for 6 h. After washing twice with PBS, the cells were collected and resuspended in 1% BSA‐containing PBS. Fluorescence intensity was analyzed using a flow cytometer (CyFlow Cube 6, Sysmex, Kobe, Japan), counting at least 5000 cells per sample.

### 4.5 Lysosomal Colocalization Study

Colon26 and RAW264.7 cells were seeded onto glass‐bottom dishes and incubated with DPPC‐CNH/ICG or IDP1‐CNH/ICG NPs under the same conditions used for the cellular uptake experiments. After incubation, the cells were washed three times with PBS and stained with LysoTracker Red DND‐99 according to the manufacturer’s protocol to visualize lysosomes. Cell nuclei were counterstained with Hoechst 33258. Fluorescence images were acquired using a fluorescence microscope (BZ‐X800; Keyence Corporation, Tokyo, Japan). NP localization was visualized through the intrinsic fluorescence of ICG. Colocalization between NP‐associated fluorescence and lysosomal signals was qualitatively evaluated from merged fluorescence images.

### 4.6 Photothermal Conversion Measurement

A GI‐POF rigid endoscope developed by AIR WATER INC. was used for fluorescence imaging and contact‐mode laser irradiation [38, 42]. The GI‐POF rigid endoscope consisted of a 0.5 mm diameter PMMA gradient‐index lens integrated into a stainless steel endoscope body (outer diameter: 1.18 mm; effective length: 60 mm) and was compatible with insertion through a standard 16G needle. For fluorescence imaging, a 785 nm laser diode was employed, whereas an 808 nm laser diode was used for photothermal treatment. The laser irradiation fiber had an outer diameter of 0.95 mm and a laser emission area of 0.33 mm^2^ at the endoscope tip. The output power at the tip of the endoscope was adjusted to 300 or 500 mW for photothermal experiments.

For contact‐mode laser irradiation, the endoscope and thermocouple (AD‐5601A; A and D, Tokyo, Japan) were inserted into 1.8 mL glass vials equipped with a septum rubber containing IDP1‐CNH/ICG solution (0.2 mL). The 808 nm NIR laser was irradiated through the GI‐POF rigid endoscope for 5 min, and the solution temperature was measured every minute. Thermographic images were recorded using an infrared thermograph (i7; FLIR, Nashua, NH, USA).

For noncontact‐mode laser irradiation, an 808 nm NIR laser (Civil Laser, Hangzhou, Zhejiang, China) was used. IDP1‐CNH/ICG solution (0.2 mL) in 96‐well plates was irradiated with an 808 nm NIR laser, and solution temperature was measured every minute.

### 4.7 Blood Concentration Profile of ICG and In Vivo Fluorescent Imaging

Animal experiments followed all protocols approved by the Institutional Animal Care and Use Committee of JAIST (No. 04–007). Female BALB/cCrSlc mice (6 weeks old; *n* = 4; average weight = 18 g, Japan SLC) were intravenously administered with 200 μL of ICG, ICG‐MeO‐PEG, ICG‐XTEN, and ICG‐IDP1 (0.1 mg mL^−1^ ICG). At prescribed time periods, 200 μL of blood was collected using the submandibular vein method. Blood was immediately centrifuged at 3000 rpm for 5 min to collect plasma. The fluorescence intensity of fivefold diluted plasma in PBS was measured (Ex: 780 nm, Em: 800 nm). The ICG concentration was calculated from a calibration curve based on the ICG concentration and fluorescence intensity. The blood half‐life (*t*_1/2_) was estimated from the concentration–time profiles by nonlinear least‐squares curve fitting. Mice were visualized using an in vivo fluorescence imaging system with a 3 s exposure time and an ICG filter (excitation, 740–790 nm; emission, 810–860 nm). After 24 h, the mice were euthanized, and organs including the heart, liver, spleen, kidneys, and tumors were extracted and imaged. Fluorescence images were acquired and analyzed using CleVue software (Vieworks). For quantitative analysis of ex vivo fluorescence images, regions of interest (ROIs) were manually defined for each harvested organ and tumor using CleVue software. Mean fluorescence intensity was calculated from the ROI area after background subtraction. Data are presented as mean ± standard deviation (SD) from three independent animals. Background fluorescence was determined from a nonfluorescent region of the image and subtracted from each ROI measurement.

### 4.8 In Vivo Anticancer Therapy

All animal experiments strictly followed all protocols approved by the Institutional Animal Care and Use Committee of the JAIST (Approval No. 07–009) and followed the Animal Research: Reporting of In Vivo Experiments guidelines. Female BALB/cCrSlc mice (*n* = 5 per group; 6 weeks old; average weight = 18 g) were administered with 1 × 10^6^ Colon26 cells in 200 μL Matrigel culture medium (v/*v* = 1:1; Dow Corning, Corning, NY, USA) into the dorsal right side to generate Colon26‐bearing mice. After approximately 1 week, when tumor volume reached approximately 100 mm^3^, mice were intravenously injected with 200 μL saline or IDP1‐CNH/ICG (0.1 mg mL^−1^ CNH/ICG). Experiments with and without laser irradiation were performed for each group. At 24 h post‐administration, tumors were irradiated with the GI‐POF rigid endoscope through a 16G needle (contact‐mode laser) or 808 nm NIR laser (noncontact‐mode laser) for 5 min. Thermographic measurements were conducted during irradiation using infrared thermography. Tumor volume and overall health (viability and body weight) were monitored every alternate day. Tumor volumes were calculated using the formula V = L × W^2^ × 0.5, where L and W denote tumor length and width, respectively. When tumor volume exceeded 1500 mm^3^, the mice were euthanized according to JAIST Institutional Animal Care and Use Committee guidelines.

### 4.9 Real‐Time Fluorescence Imaging via GI‐POF Rigid Endoscope

For image‐guided laser delivery, the GI‐POF rigid endoscope system was equipped with a coaxial fluorescence imaging module (excitation 785 nm, emission 810–860 nm). Colon26‐bearing mice were intravenously administered with IDP1‐CNH/ICG (200 μL, 100 μg mL^−1^ CNH/ICG). At 24 h post‐administration, the GI‐POF rigid endoscope was inserted into tumor tissue through a 16G needle under anesthesia. Real‐time fluorescence images were captured using the integrated camera system and recorded on a display monitor. Images were acquired at 10 s intervals during insertion to visualize ICG distribution within tumor tissue.

### 4.10 Safety Tests

The CBC was measured using a Celltac α blood cell counting machine (Microsemi LC‐712; HORIBA, Japan). BALB/cCrSlc mice (female; 6 weeks; *n* = 5; average weight = 18 g; Japan SLC, Inc.) were injected into the tail vein with saline (200 μL), IDP1 (200 μL, 100 μg mL^−1^), or IDP1‐CNH/ICG suspension (200 μL, 100 μg mL^−1^ CNH/ICG). Blood samples were collected from the inferior vena cava of each mouse after 7 days.

Colon26‐bearing female BALB/cCrSlc mice (6 weeks; average weight = 18 g; average tumor size = 100 mm^3^; Japan SLC) with laser irradiation were euthanized after 6 h. Tissues were harvested and stored in 4% paraformaldehyde (FUJIFILM Wako Pure Chemical) for H and E and immunohistochemical (IHC) staining. Analysis was performed by the Biopathology Institute Co., Ltd. (Oita, Japan) using standard protocols. Primary tumors were fixed in 10% formalin, processed for paraffin embedding, and cut into 3–4 μm thick sections. Sections were stained with hematoxylin and examined by light microscopy (IX73; Olympus, Tokyo, Japan) after incubation with primary antibody (anti‐digoxigeninperoxidase: Merck Millipore S7100, cleaved caspase‐3: Cell Signaling Technology 9661S). Areas showing positive staining in tumor tissues were analyzed using a light microscopy system (BZ‐X810; Keyence, Tokyo, Japan) with a hybrid cell count and microcell count software (Keyence).

### 4.11 Statistical Analysis

Data are expressed as the mean ± SD of at least three independent trials. Significance of differences between individual group means was assessed by one‐way analysis of variance, followed by the Tukey–Kramer multiple comparison test using OriginPro ver. 2022 (Northampton, MA, USA). Statistical significance was set at *p* < 0.05. Curve fitting was performed using nonlinear least squares fitting using OriginPro ver. 2022.

## Funding

This work was supported by the Japan Science and Technology Agency (JPMJSF2318); Japan Society for the Promotion of Science (23K17209, 23H00551, 25K21827, JPJS00420230006); Ministry of Education, Culture, Sports, Science and Technology (JPMXS0440200021).

## Conflicts of Interest

H.Y. and A.T. are employees of AIR WATER INC. This study was conducted as part of a collaborative research project with AIR WATER INC., which provided experimental and analytical support. However, the company was not involved in the interpretation of results or in the decision to publish. The other authors declare no competing interests.

## Supporting information

Supplementary Material

Supporting_Video_S1

## Data Availability

The data that support the findings of this study are available in the supplementary material of this article.
