# Supplementary material for: Intrinsically Disordered Polypeptides‐Based Stealth Materials Enable Enhanced Photothermal Cancer Therapy Using an In Situ Fiber‐Based Penetrating Laser System
Source: Small Sci. 2026 Jul 14;6(7):e70342. doi: 10.1002/smsc.70342 (PMC13387302; doi:10.1002/smsc.70342)
Supplement: Supplementary file 1 — Supplementary Material [file SMSC-6-e70342-s001.pdf]

WILEY-VCH

Supporting Information

Intrinsically Disordered Polypeptides-Based Stealth Materials Enable Enhanced Photothermal Cancer Therapy Using an In Situ Fiber-Based Penetrating Laser System

*Kei Nishida, Hiromasa Yamashita, Akira Takahashi, and Eijiro Miyako**

Reagents

Tokyo Chemical Industry (Tokyo, Japan) supplied indocyanine green (ICG) carboxylic acid, *N*-(2-aminoethyl)maleimide hydrochloride, N, N-diisopropylethylamine (DIPEA), 1, 2-Dipalmitoyl-sn-glycero-3-phosphocholine (DPPC), 1, 2-Dipalmitoyl-sn-glycero-3-phosphoethanolamine (DPPC-amine), trifluoroacetic acid (TFA), and *N*-succinimidyl 6-maleimidohexanoate. Fujifilm Wako Pure Chemical (Osaka, Japan) and NOF Corporation (Tokyo, Japan) supplied 4-(4, 6-Dimethoxy-1, 3, 5-triazin-2-yl)-4-methylmorpholinium chloride (DMT-MM) and α-Methoxy-ω-mercapto poly(ethylene glycol) (MeO-PEG-SH, M_w 5,000), respectively. All other solvents and reagents were purchased from Fujifilm Wako Pure Chemicals and Nacalai Tesque (Kyoto, Japan).

Synthesis of maleimide-conjugated ICG (Mal-ICG) (Scheme S1)

ICG carboxylic acid (209 mg, 0.256 mmol), *N*-(2-aminoethyl)maleimide (50 mg, 0.284 mmol), DMT-MM (78.3 mg, 0.284 mmol), and DIPEA (36.7 mg, 0.284 mmol) were dissolved in dehydrated methanol (10 mL), and the solution was stirred for 16 h at 4°C under dark conditions. After the reaction, the solution was evaporated, and the residual was dissolved in dichloromethane (CH_2Cl_2). The solution was extracted with brine and water. The evaporated sample was purified by silica column chromatography (CH_2Cl_2 : hexane = 8:2). The recovered solution was evaporated to obtain Mal-ICG (58.2 mg, 22.6% yield). ^{1}H NMR spectra (DMSO-*d*$_6$) were recorded on a Bruker Avance III 400 MHz spectrometer (Bruker BioSpin, Rheinstetten, Germany) (**Figure S9**).

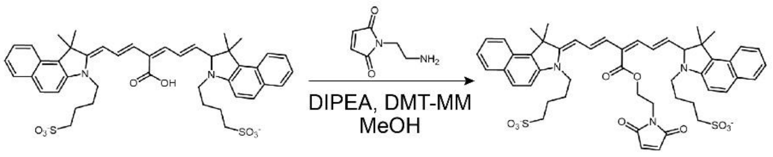

Scheme S1 Synthesis of maleimide-conjugated ICG (Mal-ICG)

Synthesis of maleimide group-conjugated DPPC (Mal-DPPC) (Scheme S2)

DPPC-amine (200 mg, 0.272 mmol), *N*-succinimidyl 6-maleimidohexanoate (165.2 mg, 0.536 mmol), and DIPEA (112.0 μL, 1.34 mmol) dissolved in dehydrated chloroform (20 mL) were stirred for 16 h at 4°C under vacuum conditions. After the reaction, the solution was evaporated and purified by silica column chromatography (CH_2Cl_2 : hexane = 10 : 3). The recovered solution was evaporated to obtain Mal-DPPC (75.8 mg, 31.5% yield). ^{1}H NMR spectra (DMSO-*d*$_6$) were recorded on a Bruker Avance III 400 MHz spectrometer (Bruker BioSpin, Rheinstetten, Germany) (**Figure S10**).

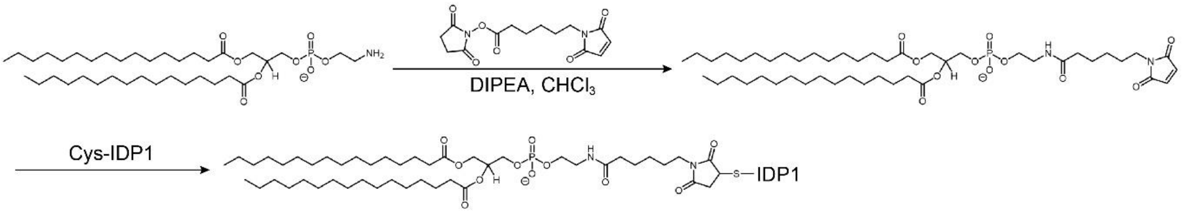

Scheme S2 Synthesis of maleimide group-conjugated DPPC (Mal-DPPC)

Conjugation of IDP1 with ICG and DPPC

IDP1 (2.64 mg, 0.174 μmol) dissolved in PBS (4 mL) was mixed with Mal-ICG (2 mg, 2.0 μmol) or Mal-DPPC (5.14 mg, 5.10 mol) dissolved in DMSO. The solutions were gently stirred at 4°C for 18 h. The reacted solutions were dialyzed (MWCO: 3,500) against PBS containing 20% methanol and PBS to obtain ICG-IDP1 and DPPC-IDP1. XTEN and MeO-PEG-SH were also reacted with Mal-ICG and Mal-DPPC in the same procedure. Samples were stored at −20°C. Protein concentration and absorbance of the ICG group were determined using a Pierce BCA Protein Assay Kit and UV–VIS–NIR spectrometry (V-730 BIO; Jasco, Tokyo, Japan). Conjugation of ICG groups to polypeptides was confirmed by visualization of SDS-PAGE gels using an in vivo fluorescence imaging system (VISQUE InVivo Smart-LF, Vieworks, Anyang, Korea) and CBB staining (**Figure S7**). Conjugation of the ICG groups to IDP1 was confirmed by HPLC (JASCO) equipped with TSKgel Octadecyl-4PW (7 µm column, 4.6 mm × 15 cm, Tosoh) at 220 nm (**Figure S8**). HPLC required gradient conditions (from 0% solvent A to 40% acetonitrile over 30 min) with a flow rate of 0.5 mL min^{-1}. Solvent A: water containing 0.2% TFA, B: acetonitrile containing 0.2% TFA.

Viability of cells treated with IDP1-CNH/ICG

Colon26 and MRC5 cells were obtained from the Japanese Collection of Research Bioresources Cell Bank (Tokyo, Japan). RAW264.7 cells were obtained from ATCC (Manassas, VA, USA). Colon26, MRC5, and RAW264.7 cells were cultured in Dulbecco's Modified Eagle's Medium (Gibco, Grand Island, NY, USA) supplemented with 10% FBS, 2 mM L-glutamine, 1 mM sodium pyruvate, gentamycin, and 100 IU mL^{-1} penicillin-streptomycin. The cells were maintained at 37°C in a humidified chamber containing 5% CO_2. Cells were seeded on a 96-well plate at 1×10^4 cells cm^{-2}, and incubated for 24 h. IDP1-CNH/ICG suspension was treated to cells at arbitrary concentration for 1 h. The NIR laser at 808 nm was irradiated using a GI-POF rigid endoscope for 2 min (300 mW). After washing twice with PBS, the cells were incubated for 24 h. Cell viability was measured using a cell-counting kit 8 (Dojindo).

Table S1 Complete blood counts (CBCs) of mice administered saline, IDP1, and IDP1-CNH/ICG.

Measured value	Entry	Unit	Saline	IDP1	IDP1-CNH/ICG	P value
CBC	WBC	$\times 10^2\ \mu L^{-1}$	70±19.4	65.25±10.8	72.5±8.10	>0.05
	RBC	$\times 10^4\ \mu L^{-1}$	1174.7±64.9	1014.5±142.6	1062.7±104.9	>0.05
	Hgb	$g\ dL^{-1}$	19.5±1.08	17.225±2.86	18.25±1.69	>0.05
	Hct	%	58.55±3.90	50.425±8.29	53.125±5.34	>0.05
	MCV	fL	49.9±0.84	49.625±1.41	49.975±0.09	>0.05
	MCH	pg	16.675±0.22	16.925±0.49	17.2±0.21	>0.05
	MCHC	$g\ dL^{-1}$	33.375±0.43	34.125±0.22	34.4±0.42	>0.05
	PLT	$\times 10^4\ \mu L^{-1}$	101.05±23.2	111.325±20.9	107.675±16.8	>0.05

Data presented as mean ± SD (n = 4 for CBC). Statistical analyses comprise the Tukey-Kramer multiple comparison test.

Abbreviations: HGB, hemoglobin; MCH, mean corpuscular hemoglobin; MCHC, mean corpuscular hemoglobin concentration; MCV, mean corpuscular volume; PLT, platelet; RBC, red blood cell; WBC, white blood cell.

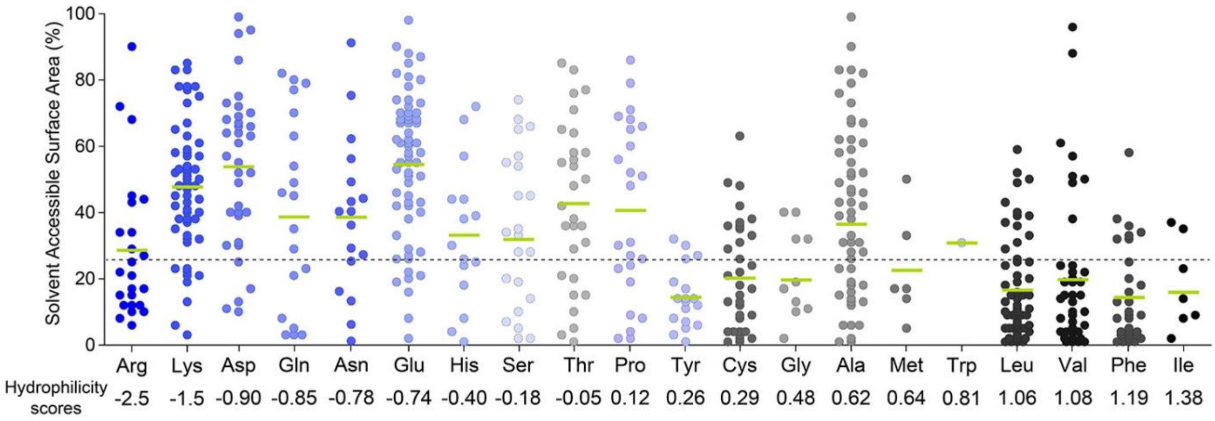

Figure S1. Average SASA value for each amino acid in HSA (PDB 1e78), calculated using PyMOL. Hydrophilicity scores of amino acids were the consensus scale by Eisenberg et al.

XTEN

GSPAGSPTSTEEGTSESATPESGPGTSTEPSEGSAPGSPAGSPTSTEEGTST
EPSEGSAPGTSTEPSEGSAPGTSESATPESGPGSEPATSGSETPGSEPATSG
SETPGSPAGSPTSTEEGTSESATPESGPGTSTEPSEGSAP

Figure S2 The amino acid sequences of XTEN.

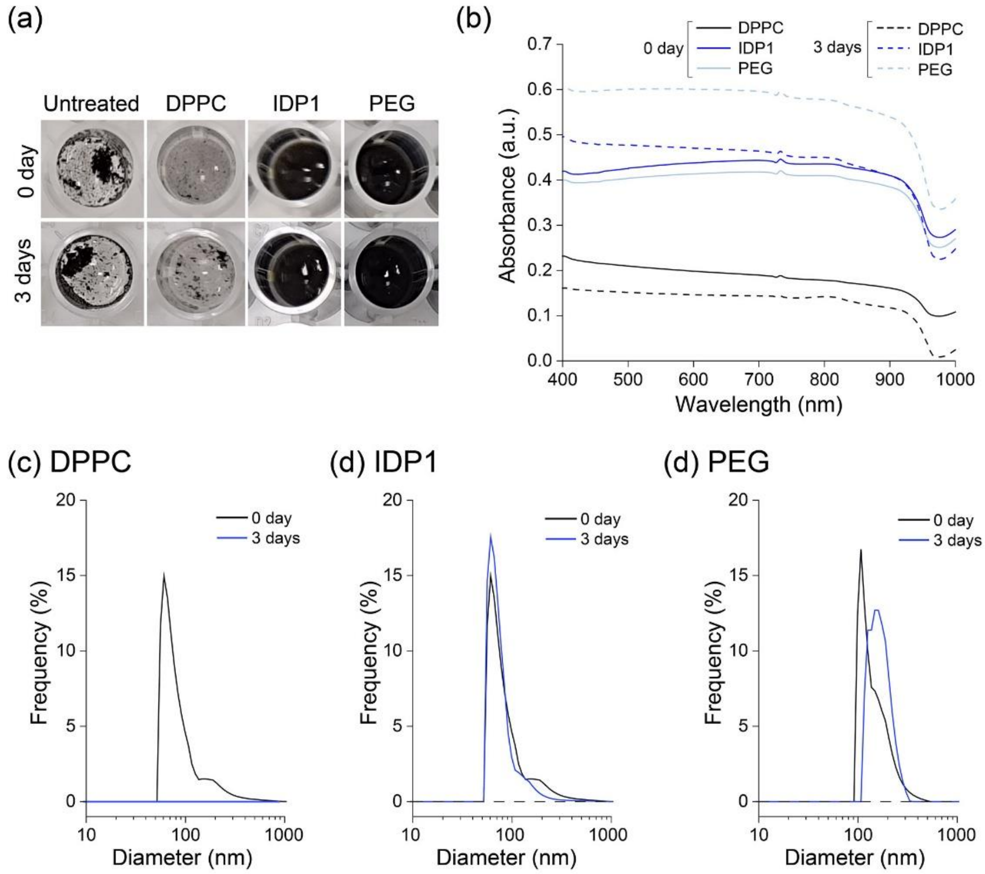

Figure S3 (**a, b**). Photograph (a) and UV–VIS–NIR absorbance spectra (b) of CNH alone, DPPC-CNH, DPPC-IDP1-CNH, DPPC-MeO-PEG-CNH after 0–3 days. **(c-d)** DLS size distribution profiles of DPPC-CNH (c), DPPC-IDP1-CNH (d), and DPPC-MeO-PEG-CNH at 0 and 3 days.

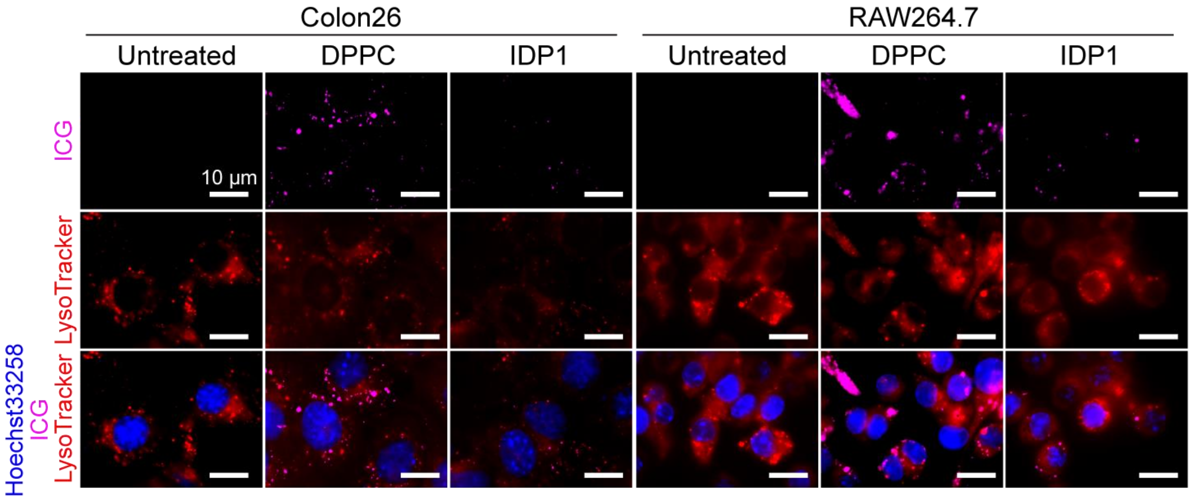

Figure S4 Lysosomal colocalization of DPPC-CNH/ICG and IDP1-CNH/ICG nanoparticles in Colon26 and RAW264.7 cells. Fluorescence microscopy images showing nanoparticle-associated ICG fluorescence (pink), lysosomes stained with LysoTracker Red (red), and nuclei stained with Hoechst 33258 (blue). Compared with DPPC-CNH/ICG nanoparticles, IDP1-CNH/ICG nanoparticles exhibited markedly lower intracellular fluorescence signals in both Colon26 and RAW264.7 cells, indicating reduced cellular internalization. The internalized nanoparticles showed substantial colocalization with lysosomal signals in merged images, suggesting localization within endo/lysosomal compartments following uptake. Scale bars: 10 μm.

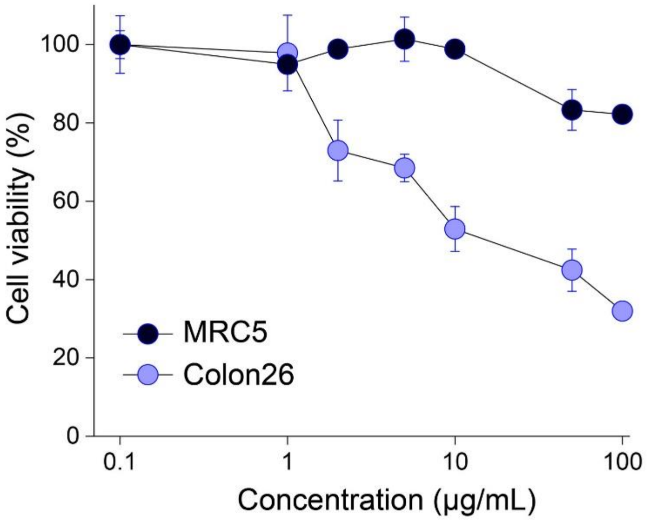

Figure S5. Viability of MRC5 and Colon26 cells treated with IDP1-CNH/ICG suspension at various concentrations of CNH/ICG, followed by NIR irradiation with a contact-mode laser (300 mW, 2 min). Data are presented as means ± SD (n = 3).

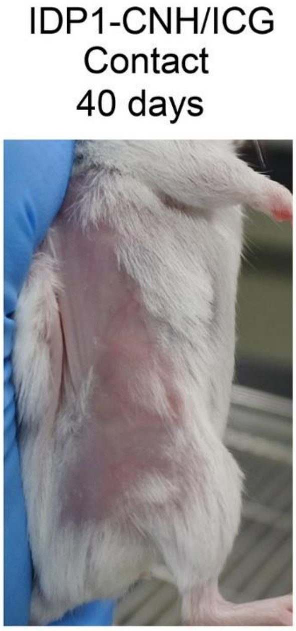

Figure S6. Representative photographs of tumor-bearing mice treated with IDP1-CNH/ICG and a contact-mode laser after 40 days.

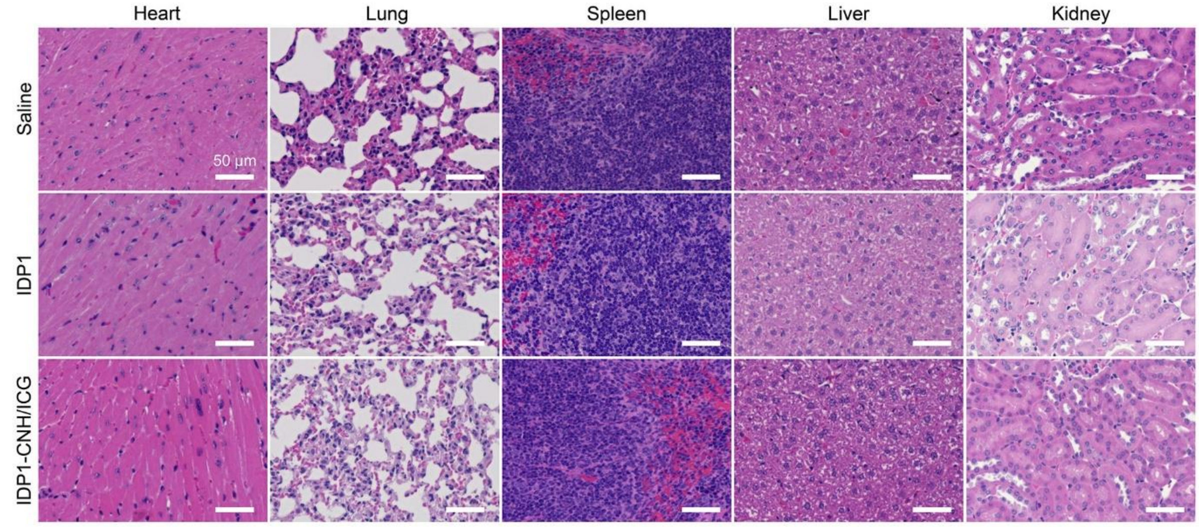

Figure S7. H and E staining in conventional organs of mice after intravenous administration with saline, IDP1 (200 μL, 100 μg mL^{-1}), and IDP1-CNH/ICG (200 μL, 100 μg mL^{-1} CNH/ICG) after 7 days. Scale bars: 50 μm.

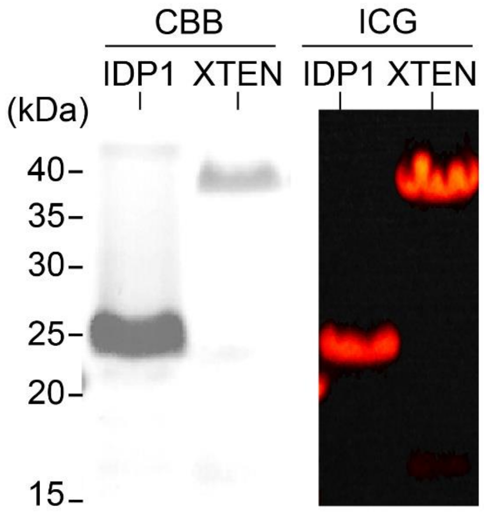

Figure S8. SDS-PAGE analysis of recombinant ICG-IDP1 and ICG-XTEN. The acrylamide gel was visualized with an NIR fluorescence imaging system (excitation 740–790 nm, emission 810–860 nm, 1 s exposure), followed by staining with CBB.

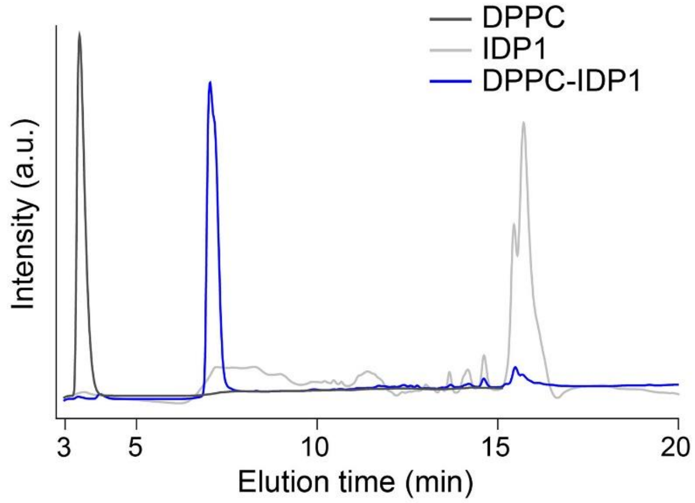

Figure S9. Reverse-phase HPLC charts of DPPC, IDP1, and DPPC-IDP1

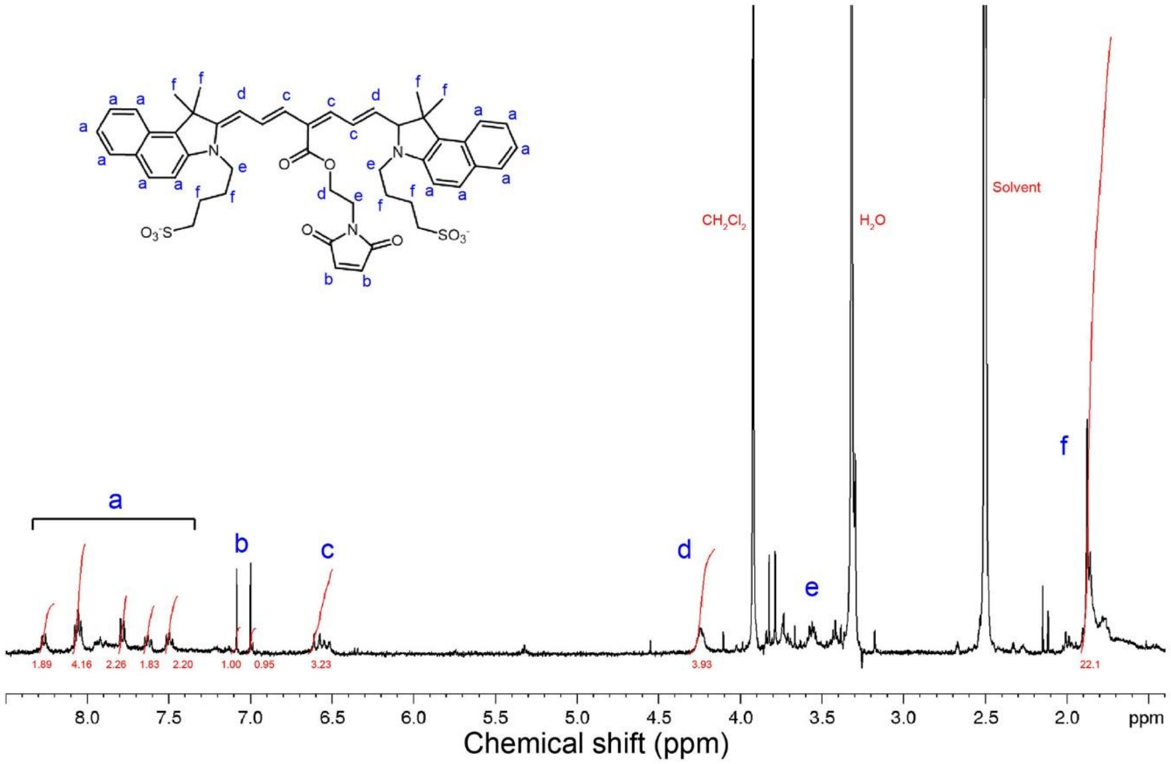

Figure S10. ^{1}H NMR spectrum of Mal-ICG (400 Hz, DMSO-d6)

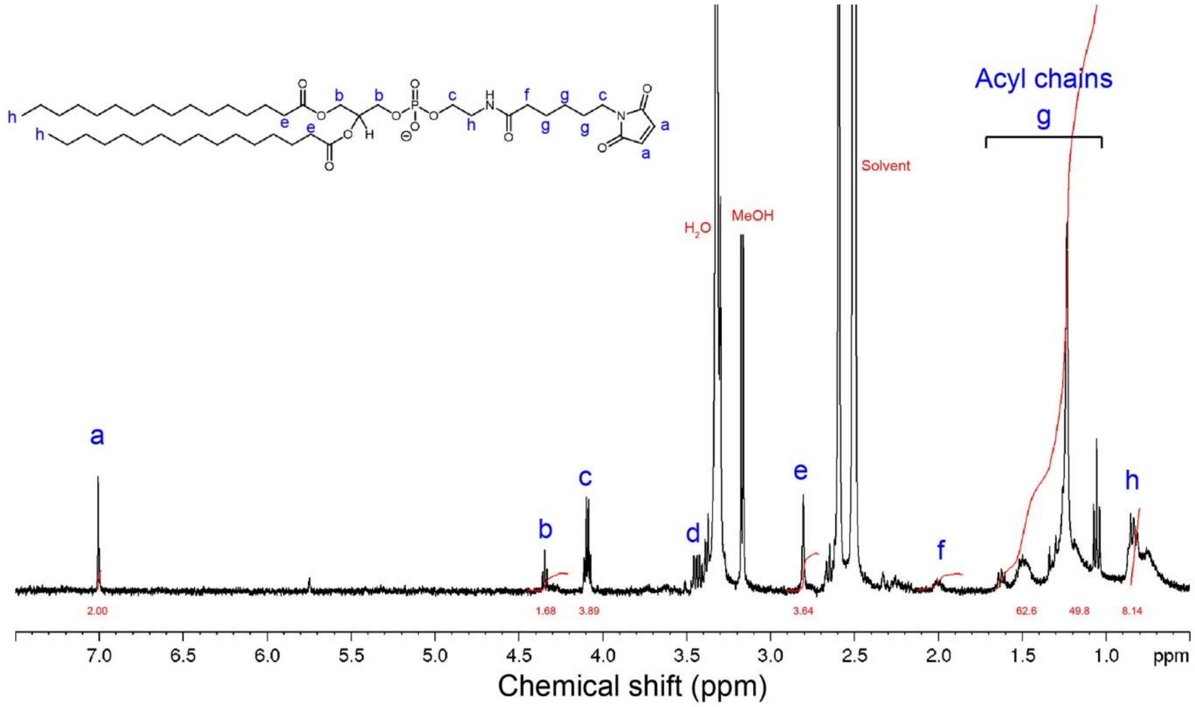

Figure S11. ^{1}H NMR spectrum of Mal-DPPC (400 Hz, DMSO-d6)

Supporting Video Legends

Supporting Video S1. Real-time fluorescence imaging capability of the GI-POF rigid endoscope system for image-guided photothermal therapy
